# In vitro structure–activity relationships and forensic case series of emerging 2-benzylbenzimidazole ‘nitazene’ opioids

**DOI:** 10.1007/s00204-024-03774-7

**Published:** 2024-06-14

**Authors:** Liam M. De Vrieze, Sara E. Walton, Eline Pottie, Donna Papsun, Barry K. Logan, Alex J. Krotulski, Christophe P. Stove, Marthe M. Vandeputte

**Affiliations:** 1 Laboratory of Toxicology, Department of Bioanalysis, Faculty of Pharmaceutical Sciences, Ghent University, Ghent, Belgium; 2 Center for Forensic Science Research and Education, Fredric Rieders Family Foundation, Willow Grove, PA 19090 USA; 3 NMS Labs, Horsham, PA 19044 USA

**Keywords:** New synthetic opioids (NSOs), 2-Benzylbenzimidazole ‘nitazene' opioids, Forensic toxicology, μ-Opioid receptor (MOR), Bioassay

## Abstract

2-Benzylbenzimidazole ‘nitazene’ opioids are presenting a growing threat to public health. Although various nitazenes were previously studied, systematic comparisons of the effects of different structural modifications to the 2-benzylbenzimidazole core structure on μ-opioid receptor (MOR) activity are limited. Here, we assessed in vitro structure–activity relationships of 9 previously uncharacterized nitazenes alongside known structural analogues. Specifically, we focused on MOR activation by ‘ring’ substituted analogues (i.e., *N*-pyrrolidino and *N*-piperidinyl modifications), ‘desnitazene’ analogues (lacking the 5-nitro group), and *N*-desethyl analogues. The results from two in vitro MOR activation assays (β-arrestin 2 recruitment and inhibition of cAMP accumulation) showed that ‘ring’ modifications overall yield highly active drugs. With the exception of 4′-OH analogues (which are metabolites), *N*-pyrrolidino substitutions were generally more favorable for MOR activation than *N*-piperidine substitutions. Furthermore, removal of the 5-nitro group on the benzimidazole ring consistently caused a pronounced decrease in potency. The *N*-desethyl modifications showed important MOR activity, and generally resulted in a slightly lowered potency than comparator nitazenes. Intriguingly, *N*-desethyl isotonitazene was the exception and was consistently more potent than isotonitazene. Complementing the in vitro findings and demonstrating the high harm potential associated with many of these compounds, we describe 85 forensic cases from North America and the United Kingdom involving etodesnitazene, *N*-desethyl etonitazene, *N*-desethyl isotonitazene, *N*-pyrrolidino metonitazene, and *N*-pyrrolidino protonitazene. The low-to-sub ng/mL blood concentrations observed in most cases underscore the drugs’ high potencies. Taken together, by bridging pharmacology and case data, this study may aid to increase awareness and guide legislative and public health efforts.

**Supplementary Information:**

The online version contains supplementary material available at 10.1007/s00204-024-03774-7.

## Introduction

**Fig. 1:**
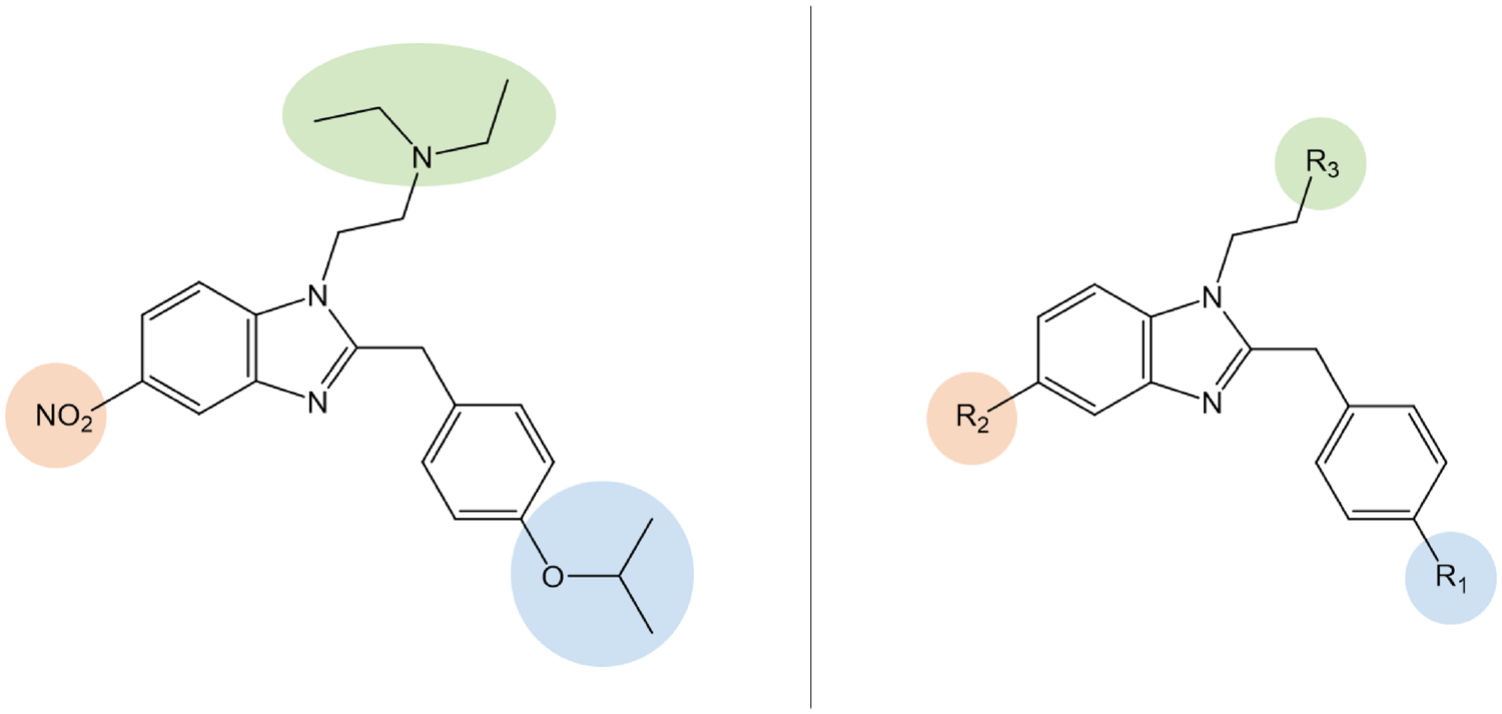
Structure of isotonitazene (left) and generic structure of 2-benzylbenzimidazole ‘nitazene’ opioids (right). The highlighted R groups correspond to the substitutions as indicated in Table 1 (color figure online)

In recent years, the availability of new synthetic opioids (NSOs) on the recreational drug market has been rising, making NSOs one of the fastest growing groups of new psychoactive substances (NPS) worldwide (UNODC 2022a; EMCDDA 2023). The presence of NSOs has further exacerbated the ongoing opioid epidemic in the United States (U.S.), where 88% of opioid-related overdose deaths involved synthetic opioids in 2021 (CDC 2023). NSOs are also increasingly identified in Europe, with a total of 74 different drugs monitored by the European Union Early Warning System (EWS) on NPS between 2009 and 2022 (EMCDDA 2023). Opioids with diverse chemical structures have emerged in response to class-wide bans on fentanyl analogues in the U.S. and China (Bao et al. 2019; UNODC 2020; 117th Congress 2021), and NSOs with a 2-benzyl-benzimidazole core (Fig. 1) have become the most dominant class of non-fentanyl-related opioids in the last 4 years (Ujváry et al. 2021; Vandeputte et al. 2021; Krotulski et al. 2023).

In the late 1950s, the Swiss pharmaceutical company *Chemische Industrie Basel* (CIBA) synthesized a series of 2-benzylbenzimidazole drugs, now commonly referred to as ‘nitazenes’, as potential new analgesics. A mouse tail-flick assay revealed that the antinociceptive potency of etonitazene, the prototypical drug in the series and the previously described most potent analogue, was 1000-fold higher than that of morphine (Gross and Turrian 1957; Hunger et al. 1957, 1960a, b). Despite their promising antinociceptive properties, nitazenes were never marketed, possibly due to a high risk of adverse effects (Bromig 1958; Ujváry et al. 2021).

**Fig. 2:**
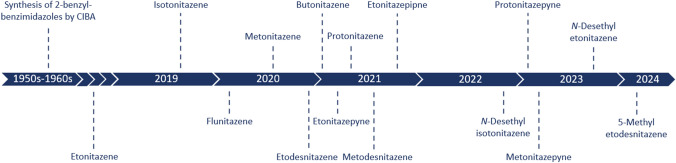
Timeline of the appearance of nitazenes on the recreational drug market in the United States (U.S.). With the exception of etonitazene (which was reported in the U.S. in 2003 but not since Reavy 2003; Morris 2009)) and 5-methyl etodesnitazene (Krotulski et al. 2024), dates indicate the date of receipt of forensic case samples and/or drug materials at the Center for Forensic Science Research and Education (CFSRE) (color figure online)

Apart from occasional identifications of etonitazene between 1966 and 2003 (Brandenberger 1974; Sorokin 1999; Sorokin et al. 1999; Reavy 2003; Morris 2009), it was not until 2019 that the first nitazene was identified on the recreational drug market (Blanckaert et al. 2020; Vandeputte et al. 2021). That drug was isotonitazene (Figs. 1, 2), which quickly populated the NSO market and has since been implicated in hundreds of deaths worldwide, stressing the dangerous nature of this family of opioids (EMCDDA 2020; Krotulski et al. 2020; Mueller et al. 2021; Vandeputte et al. 2022a; Montanari et al. 2022). Following the introduction of legislations targeting isotonitazene (EC 2020; UNODC 2021; DEA 2021), various other nitazenes were identified on recreational drug markets and eventually scheduled. Currently, a number of nitazenes are subject to international control (i.e., etonitazene, clonitazene, isotonitazene, metonitazene, protonitazene, butonitazene, etodesnitazene (‘etazene’), and *N*-pyrrolidino etonitazene (‘etonitazepyne’)) (UNODC 1975, 2022b, 2023). In the U.S., different nitazenes such as *N*-piperidinyl etonitazene (‘etonitazepipne’), *N*-desethyl isotonitazene, metodesnitazene, and flunitazene were individually scheduled and are temporarily designated as Schedule I substances by the U.S. Drug Enforcement Administration (DEA) (DEA 2022, 2023). At the time of writing (Q2 2024), 15 nitazenes have been identified in the U.S., with the latest additions being *N*-pyrrolidino metonitazene (‘metonitazepyne’), *N*-pyrrolidino protonitazene (‘protonitazepyne’), *N*-desethyl etonitazene, and 5-methyl etodesnitazene ('etomethazene') (Fig. 2).

The chemical structures of the hitherto identified 2-benzylbenzimidazole opioids vary in three positions of the molecule (Fig. 1): (a) the *para*-benzyl position, with typically either a differing alkoxy chain length (e.g., meto-, eto-, proto-, isoto-, and butonitazene) or halogen substitution (e.g., flunitazene) (R_1_); (b) the 5-position of the benzimidazole ring containing or lacking a nitro moiety (e.g., etodesnitazene vs. etonitazene) (R_2_); and (c) the substituted ethyl amino side chain attached to the benzimidazole ring, typically containing a tertiary amine with an *N*,*N*-diethyl moiety (R_3_). Examples of modifications of the latter include *N*-desethyl analogues (e.g., *N*-desethyl etonitazene and *N*-desethyl isotonitazene) and ‘ring’ analogues with either a pyrrolidine (e.g., *N*-pyrrolidino etonitazene (‘etonitazepyne’)) or piperidine substitution (e.g., *N*-piperidinyl etonitazene (‘etonitazepipne’)).

While various nitazenes have been characterized both in vitro and in vivo for their opioid activity (Ujváry et al. 2021; Vandeputte et al. 2021, 2022b, e, 2023; De Luca et al. 2022; Kanamori et al. 2023; Walton et al. 2023; Malcolm et al. 2023; Glatfelter et al. 2023; Kozell et al. 2024; Tsai et al. 2024), the continuing emergence of new analogues (e.g., *N*-pyrrolidino metonitazene and *N*-pyrrolidino protonitazene) for which no or only limited pharmacology data are available, stresses the need for up-to-date reporting and evaluation of this class of NSOs. Furthermore, systematic comparisons of the impact of different structural alterations to the nitazene core structure (Fig. 1) on µ-opioid receptor (MOR) activity are limited, specifically when considering different ‘ring’ substitutions (R_3_ position in Fig. 1). This study focused on the in vitro functional characterization of a comprehensive set of 25 differentially substituted nitazenes at MOR, using a β-arrestin 2 recruitment assay (as a measure of G protein-independent pathway activation) and an intracellular cyclic adenosine monophosphate (cAMP) assay (as a measure of the G_αi_ protein pathway activation). Of the studied drugs, 9 analogues were not previously characterized. In addition, we bridged the pharmacology data with case data and report the first identifications of etodesnitazene, *N*-desethyl etonitazene, *N*-desethyl isotonitazene, *N*-pyrrolidino metonitazene, and *N*-pyrrolidino protonitazene in forensic toxicology casework from North America, as well as the detection of *N*-pyrrolidino protonitazene, *N*-pyrrolidino metonitazene, and *N*-desethyl isotonitazene in forensic cases from the United Kingdom (U.K.). The prevalence, geographical distribution, and toxicological findings are discussed.

## Materials and methods

### Materials

Hydromorphone (**A**), morphine (**B**), fentanyl (**C**), and DAMGO trifluoroacetate (**D**) were purchased from Cayman Chemical (Ann Arbor, MI, U.S.). The reference standards for metonitazene (**1**), *N*-pyrrolidino metonitazene citrate (**2**), *N*-piperidinyl metonitazene citrate (**3**), metodesnitazene hydrochloride (**4**), *N*-pyrrolidino metodesnitazene citrate (**5**), *N*-desethyl metonitazene hydrochloride (**6**), etonitazene (**7**), *N*-pyrrolidino etonitazene (**8**), *N*-piperidinyl etonitazene citrate (**9**), etodesnitazene citrate (**10**), *N*-pyrrolidino etodesnitazene citrate (**11**), *N*-desethyl etonitazene (**12**), protonitazene hydrochloride (**13**), *N*-pyrrolidino protonitazene (**14**), *N*-piperidinyl protonitazene citrate (**15**), protodesnitazene citrate (**16**), *N*-desethyl protonitazene hydrochloride (**17**), isotonitazene (**18**), *N*-pyrrolidino isotonitazene citrate (**19**), *N*-piperidinyl isotonitazene citrate (**20**), isotodesnitazene citrate (**21**), *N*-desethyl isotonitazene hydrochloride (**22**), 4′-OH nitazene (**23**), *N*-pyrrolidino 4′-OH nitazene citrate (**24**), and *N*-piperidinyl 4′-OH nitazene citrate (**25**) were kindly gifted by Cayman Chemical (Ann Arbor, MI, U.S.). Forskolin was obtained from Tocris Bioscience (Bristol, U.K.) and from Enzo Life Sciences (Farmingdale, NY, U.S.). For preparation of the stock solutions for in vitro experiments, methanol (MeOH) was obtained from Chem-Lab NV (Zedelgem, Belgium), dimethylsulfoxide (DMSO) was procured from Merck KGaA (Darmstadt, Germany), and acetonitrile (ACN) was purchased from Biosolve (Valkenswaard, Netherlands). Dulbecco’s Modified Eagle’s Medium (DMEM, GlutaMAX^®^), Opti-MEM^®^ I Reduced Serum Medium, CO_2_-independent medium, penicillin/streptomycin (10,000 IU/mL and 10,000 μg/mL), and amphotericin B (250 μg/mL) were obtained from Thermo Fisher Scientific (Pittsburgh, PA, U.S.). The Nano-Glo^®^ Live Cell Assay System (consisting of Nano-Glo^®^ Live Cell Substrate and Nano-Glo^®^ LCS Dilution Buffer), FuGENE^®^ HD transfection reagent, the pGloSensor-22F cAMP plasmid, and GloSensor^®^ cAMP reagent were purchased from Promega (Madison, WI, U.S.). Sigma-Aldrich (Overijse, Belgium) supplied fetal bovine serum (FBS) and poly-d-lysine hydrobromide. The pNBe2 expression vector encoding the human MOR, C-terminally tagged with SmBiT (part of the NanoBiT^®^ system), was kindly provided by Dr. Andy Chevigné and was previously reported by Meyrath et al. (2020). For the GloSensor^®^ cAMP assay, the cDNA sequences encoding linker-SmBiT were deleted from this construct via site-directed mutagenesis (Supplementary Information S1).

For the casework portion of this study, etodesnitazene, *N*-desethyl etonitazene, *N*-desethyl isotonitazene, *N*-pyrrolidino protonitazene, *N*-pyrrolidino metonitazene, and isotonitazene-D7 were purchased from Cayman Chemical and prepared as 1 mg/mL stock solutions in MeOH or DMSO. Ethyl acetate, *N*-butyl chloride, and LC–MS grade solvents were purchased from Honeywell Chemicals (Charlotte, NC, U.S.). Sodium borate decahydrate was purchased from Sigma-Aldrich (St. Louis, MO, U.S.) and formic acid ampules were purchased from Thermo Fisher Scientific (Waltham, MA, U.S.).

### In vitro functional characterization at the μ-opioid receptor (MOR)

Two in vitro assays were employed to determine the biological activity of a panel of 25 nitazenes and 4 reference opioids at MOR, a G protein-coupled receptor capable of activating both G_i_ proteins and β-arrestins. MOR is the main molecular target for most therapeutic and abused opioids (Charbogne et al. 2014).

#### Cell culture and transfection

The original human embryonic kidney (HEK) 293T cell line was kindly gifted by Prof. Dr. O. De Wever (Ghent University Hospital, Belgium) and was previously modified to stably express the MOR β-arrestin 2 (βarr2) reporter system (cfr. infra). The stability of the cell line was monitored on a regular basis by means of flow cytometric analysis of co-expressed markers (Vasudevan et al. 2020). The cells were routinely cultured in DMEM (GlutaMAX^®^, supplemented with 10% heat-inactivated FBS, 100 IU/mL penicillin, 100 mg/L streptomycin, and 0.25 mg/L amphotericin B) in a humidified atmosphere at 37 °C and 5% CO_2_. For the GloSensor^®^ cAMP assay, HEK 293T cells were seeded in 6-well plates (VWR, Leuven, Belgium) at a density of 5 × 10^5^ cells/well. After overnight incubation, the cells were transfected with 1.65 μg of MOR plasmid DNA (cfr. supra) and 1.65 μg of the pGloSensor-22F cAMP plasmid, using a 3:1 ratio of FuGENE^®^ HD to DNA in Opti-MEM^®^, according to the manufacturer’s protocol. To assess the presence of non-MOR-mediated signals in the GloSensor^®^ cAMP system, HEK 293T cells were transfected with 1.65 μg of pcDNA3.1 plasmid DNA instead of MOR plasmid DNA in a separate set of experiments.

#### NanoBiT^®^ MOR β-arrestin 2 recruitment assay protocol

A previously reported, stable cell-based assay (Cannaert et al. 2018; Vasudevan et al. 2020) was used to monitor MOR-mediated G protein-independent pathway activation. Shortly, MOR activation by an agonist leads to the intracellular recruitment of βarr2 (co-expressed with G protein-coupled receptor kinase 2, GRK2). This leads to the functional complementation of a split nanoluciferase (NanoLuc^®^ Binary Technology, Promega), which results in a measurable luminescent signal upon addition of the substrate furimazine.

One day prior to the assay, cells expressing the MOR-βarr2-GRK2 assay system were seeded on poly-d-lysine-coated 96-well plates (Novolab, Geraardsbergen, Belgium) at a density of 5 × 10^4^ cells/well. Following overnight incubation, the cells were rinsed twice with Opti-MEM^®^ to clear residual FBS before adding 90 μL Opti-MEM^®^ to all wells. Thereafter, the Nano-Glo^®^ Live Cell Reagent was prepared by diluting the Nano-Glo^®^ Live Cell substrate 20-fold with Nano-Glo^®^ LCS Dilution Buffer, and 25 μL was added to each well. The plate was then placed into a TriStar^2^ LB 942 multimode microplate reader (Berthold Technologies GmbH & Co., Bad Wildbad, Germany) and luminescence was continuously monitored for 10–15 min to allow stabilization of the signal. Next, 20 μL of a 6.75-fold concentrated solution of each opioid reference standard was added to the cells and luminescence was further monitored for 2 h. The solvent used varied depending on the specific compound: Opti-MEM^®^/MeOH for compounds (**2**–**3**), (**5**–**9**), (**11**), (**13**–**25**), and (**A**–**D**); Opti-MEM^®^/ACN for compounds (**1**), (**4**), (**10**), and (**12**); and Opti-MEM^®^/MeOH/DMSO for compound (**2**). Appropriate solvent controls were included in each experiment.

#### GloSensor^®^ cAMP assay

The GloSensor^®^ cAMP assay was used to measure G protein signaling following MOR activation by monitoring G_αi_-induced inhibition of cAMP formation. At least 6 h post-transfection, cells were reseeded on poly-d-lysine-coated 96-well plates at a density of 5 × 10^4^ cells/well. Following overnight incubation, the cells were rinsed with CO_2_-independent medium. According to the manufacturer’s protocol, 100 μL of equilibration medium (CO_2_-independent medium with 2% GloSensor^®^ substrate) was added to each well, followed by a 30min incubation of the cells at 37 °C. The plate was then allowed to cool to room temperature for 15 min in the dark, after which it was placed into a TriStar^2^ LB 942 multimode microplate reader for a 10 min continuous readout of the initial luminescence values. Next, the cells were pre-incubated for 10 min with 10 μL of 11-fold concentrated solutions of test compounds (in Opti-MEM^®^/MeOH, Opti-MEM^®^/ACN or Opti-MEM^®^/MeOH/DMSO, cfr. supra). Thereafter, 10 μL of a 12-fold concentrated stock solution of the adenylate cyclase activator forskolin (in Opti-MEM^®^/DMSO; final concentration 500 nM) was added to each well to stimulate endogenous cAMP levels. The luminescence readout was then continued for 2 h. Each experiment included appropriate solvent controls.

#### Data analysis

In both assays, all compounds were tested in concentrations ranging between 0.1 pM and 100 μM in minimum three independent experiments (*n* = 3–5), with duplicates included for each concentration within an experiment. In line with earlier studies (Vasudevan et al. 2020; Vandeputte et al. 2022d), hydromorphone (**A**) was used as the reference compound for normalization; fentanyl (**B**), morphine (**C**), and DAMGO (**D**) were included as additional comparators to facilitate interpretation of the results. All the concentrations are expressed as free base concentrations.

The data analysis was kept maximally similar for both assays. The obtained time-luminescence profiles were corrected for inter-well variability before calculation of the area under the curve (AUC) (Pottie et al. 2020b). Solvent correction was achieved by subtraction of the mean AUC value of the corresponding blank. Next, concentration–response curves were generated via three-parameter nonlinear regression using GraphPad Prism 9 software (San Diego, CA, U.S.). The MOR-βarr2 data were normalized by setting ‘0%’ as 0 and setting the maximal response of hydromorphone as ‘100%’. In line with previous studies from our group (Vandeputte et al. 2020, 2021; Pottie et al. 2020a), the normalized AUC values from the highest concentration(s) of each compound were excluded in case of a reduction of 20% or more compared to the AUC of the subsequent dilution, to avoid inadvertent skewing of the concentration–response curve due to potential cellular toxicity or solubility issues. For normalization of the GloSensor^®^ cAMP data, ‘0%’ was defined as the AUC value obtained from cells treated with solvent control and 500 nM forskolin, while ‘100%’ was defined as the maximum response of hydromorphone. The standard Grubbs’ test was used to screen the complete dataset (1470 MOR-βarr2 data points and 1378 GloSensor^®^ cAMP data points) for outliers, resulting in the identification and exclusion of 8 outliers (0.54%) in the MOR-βarr2 dataset and 8 outliers (0.58%) in the cAMP dataset. The potency (EC_50_) and efficacy (*E*_max_, relative to hydromorphone) values for each compound were obtained using three-parameter logistic regression (GraphPad Prism 9) on the combined normalized data from all experiments.

Due to the unknown stoichiometry of DAMGO trifluoroacetate salt (**D**), EC_50_ values obtained from full concentration–response curves (assuming a 1:1 stoichiometry) were not computed – only the fitted *E*_max_ values (three-parameter fit) were used for further analysis. In the MOR-βarr2 assay, DAMGO was run in a separate set of experiments and a calculated efficacy is reported (Supplementary Information S2).

### Toxicological analysis of etodesnitazene, *N*-desethyl etonitazene, *N*-desethyl isotonitazene, *N*-pyrrolidino metonitazene, and *N*-pyrrolidino protonitazene

#### Sample origins

Biological specimens from medicolegal death investigation cases and drug-impaired driving cases were collected by medical examiners and coroners or law enforcement, respectively, and were sent to NMS Labs (Horsham, PA, U.S.) and/or to the Center for Forensic Science Research and Education (CFSRE) (Willow Grove, PA, U.S.) for toxicology testing. When necessary, the samples were de-identified to not include any personal identifying information. Demographic information (e.g., age, sex, city, and state) and brief case histories were available for most cases. Institutional Review Board approval was not required for this protocol as it was deemed, by definition, to not involve human subjects (Krotulski et al. 2021b).

#### Initial toxicology testing

Forensic toxicology drug screening was conducted at NMS Labs and at the CFSRE. Both routine and comprehensive toxicology screenings were executed on biological specimens to detect illicit, therapeutic, and novel synthetic drugs by NMS Labs (*n* > 300) (NMS Labs 2023) and the CFSRE (*n* > 1100) (CFSRE 2023). Screening analysis was performed by liquid chromatography time-of-flight mass spectrometry (LC-TOF-MS) with an Agilent Technologies 6230 (Santa Clara, CA, U.S.) at NMS Labs. The CFSRE employed liquid chromatography quadrupole time-of-flight mass spectrometry (LC-QTOF-MS) using a Sciex X500R and Sciex TripleTOF 5600+ (Framingham, MA, U.S.). The laboratory workflow was previously described for the identification of isotonitazene, brorphine, metonitazene, and *N*-pyrrolidino etonitazene (Krotulski et al. 2020, 2021a, b; Vandeputte et al. 2022b).

Targeted data processing was employed for etodesnitazene, *N*-desethyl etonitazene, *N*-desethyl isotonitazene, *N*-pyrrolidino metonitazene, and *N*-pyrrolidino protonitazene using Agilent MassHunter Qualitative Analysis Workflows (B.08.00), Sciex MasterView, and PeakView (Version 2.2) with their respective chemical information programmed into the software, including names, formulas (C_22_H_29_N_3_O, C_20_H_24_N_4_O_3_, C_21_H_26_N_4_O_3_, C_21_H_24_N_4_O_3_, C_23_H_28_N_4_O_3_), exact protonated masses (352.2383 Da, 369.1921 Da, 383.2078 Da, 381.1921 Da, 409.2234 Da), and retention times (NMS: 4.98, *N-desethyl etonitazene not in scope*, 5.49, 4.77, 5.62 min; CFSRE: 5.43, 6.46, 6.84, 4.26, 6.97 min) based on the analysis of standard reference material. *N*-Desethyl etonitazene was not within the scope of NMS Labs library since certified reference material had not yet been verified through testing protocols. Presumptively positive results were filtered based on mass error, retention time error, isotope score, and library match (CFSRE only). All positive results were manually reviewed by a toxicologist, which encompassed the evaluation of isotope distribution, peak shape, and chromatographic characteristics.

Samples were either received directly by the CFSRE or transferred under chain of custody from NMS Labs to the CFSRE. Biological samples from all individual cases positive for etodesnitazene (*n* = 26), *N*-desethyl etonitazene (*n* = 1), *N*-desethyl isotonitazene (*n* = 16), *N*-pyrrolidino metonitazene (*n* = 15), and *N*-pyrrolidino protonitazene (*n* = 39) were subsequently subjected to quantitative confirmation by liquid chromatography-tandem quadrupole mass spectrometry (LC-QQQ-MS) at the CFSRE.

#### Quantitation of target analytes

Quantitative determination of etodesnitazene, *N*-desethyl isotonitazene, *N*-pyrrolidino metonitazene, and *N*-pyrrolidino protonitazene in biological specimens was performed using LC-QQQ-MS employing a standard addition approach (Krotulski et al. 2020, 2021a; Fogarty et al. 2022). The analytical method for etodesnitazene was developed and validated in 2022 at the height of popularity of etodesnitazene, while the method for *N*-desethyl isotonitazene, *N*-pyrrolidino metonitazene, and *N*-pyrrolidino protonitazene was developed after the emergence of these newer nitazenes in 2023. *N*-Desethyl etonitazene underwent qualitative confirmation only using LC-QTOF-MS, as the case was only received in late 2023. Four replicate samples (0.5 mL each) were aliquoted and three were fortified with the analyte of interest and ‘up-spiked’ with 0.2, 2 and 20 ng/mL; one sample remained unfortified (‘blank’). Internal standard (isotonitazene-D7) was added to all samples at a final concentration of 10 ng/mL. Samples underwent liquid–liquid extraction (LLE) using 1 mL of borax buffer (0.1 M, pH 10.4), and 3 mL of extraction solvent comprised of *N*-butyl chloride and ethyl acetate (70:30, *v*:*v*). After drying and reconstituting in 200 μL initial chromatographic conditions (60A:40B), the samples were analyzed using a Waters Xevo TQ-S micro LC-QQQ-MS (Milford, MA, U.S.). Chromatographic separation was achieved through gradient elution using 0.1% formic acid in water (A) and 0.1% formic acid in MeOH (B) on an Agilent InfinityLab Poroshell 120 EC-C18 (3.0 × 100 mm, 2.7 μm) analytical column. The flow rate was set at 0.4 mL/min and the injection volume was 5 μL. The column temperature was 30 °C. Positive electrospray ionization (ESI+) was employed, followed by multiple reaction monitoring (MRM) for mass filtration and detection. Three transitions were monitored per analyte of interest to increase specificity (*m*/*z* 352.2 > 72.0, 100.0 and 106.9 for etodesnitazene, *m*/*z* 383.2 > 72.1, 107.1 and 312.2 for *N*-desethyl isotonitazene, *m*/*z* 381.2 > 98.1, 56.1, and 121.1 for *N*-pyrrolidino metonitazene and *m*/*z* 409.2 > 98.1, 56.1 and 107.1 for *N*-pyrrolidino protonitazene, with the respective first transition representing the quantification ion for each analyte).

Concentrations were calculated by plotting the analyte-internal standard peak area ratios against up-spike concentrations (0, 0.2, 2, and 20 ng/mL). Linear regression analysis was used to determine the correlation between the data points (*R*^2^ > 0.98). The concentration in the sample was determined through back-calculation of the x-intercept, reported as a positive number. The compiled quantitative data per analogue were generated using the quantifiable blood samples (> lower limit of quantitation (LLOQ) of 0.1 ng/mL) of individual cases. Upon availability of multiple blood concentrations of the analyte of interest within an individual case, only the peripheral blood concentration was included.

#### Qualitative prevalence and mapping of geographical distribution

Surveillance of NPS plays a crucial role in assessing a drug’s overall impact before confirmatory data from forensic laboratories or medical examiner offices become available. Surveillance programs also enable real-time identification of NPS and further data analysis of important trends. In this context, the CFSRE, through its NPS Discovery drug early warning system [www.npsdiscovery.org], in partnership with forensic toxicology laboratories including NMS Labs, has set up toxicology surveillance initiatives using continuous high-resolution mass spectrometry data. Evaluation of the data allowed the prevalence determination of etodesnitazene, *N*-desethyl etonitazene, *N*-desethyl isotonitazene, *N*-pyrrolidino metonitazene, and *N*-pyrrolidino protonitazene. This was achieved by tracking the number of positive cases each month since the initial identification of the nitazene in question. NMS Labs processes more than 100,000 cases per year that originate from most U.S. states and some Canadian territories. Samples that undergo confirmatory testing for NPS are transferred to the CFSRE for surveillance analysis. The quarterly data from CFSRE’s comprehensive toxicological analyses on postmortem cases and a driving under the influence of drugs (DUID) investigation were used to generate the figures displaying the prevalence and geographical distribution of the 5 nitazenes evaluated herein.

## Results

### Pharmacological characterization and structure–activity relationship determination of 2-benzylbenzimidazole opioids

**Fig. 3:**
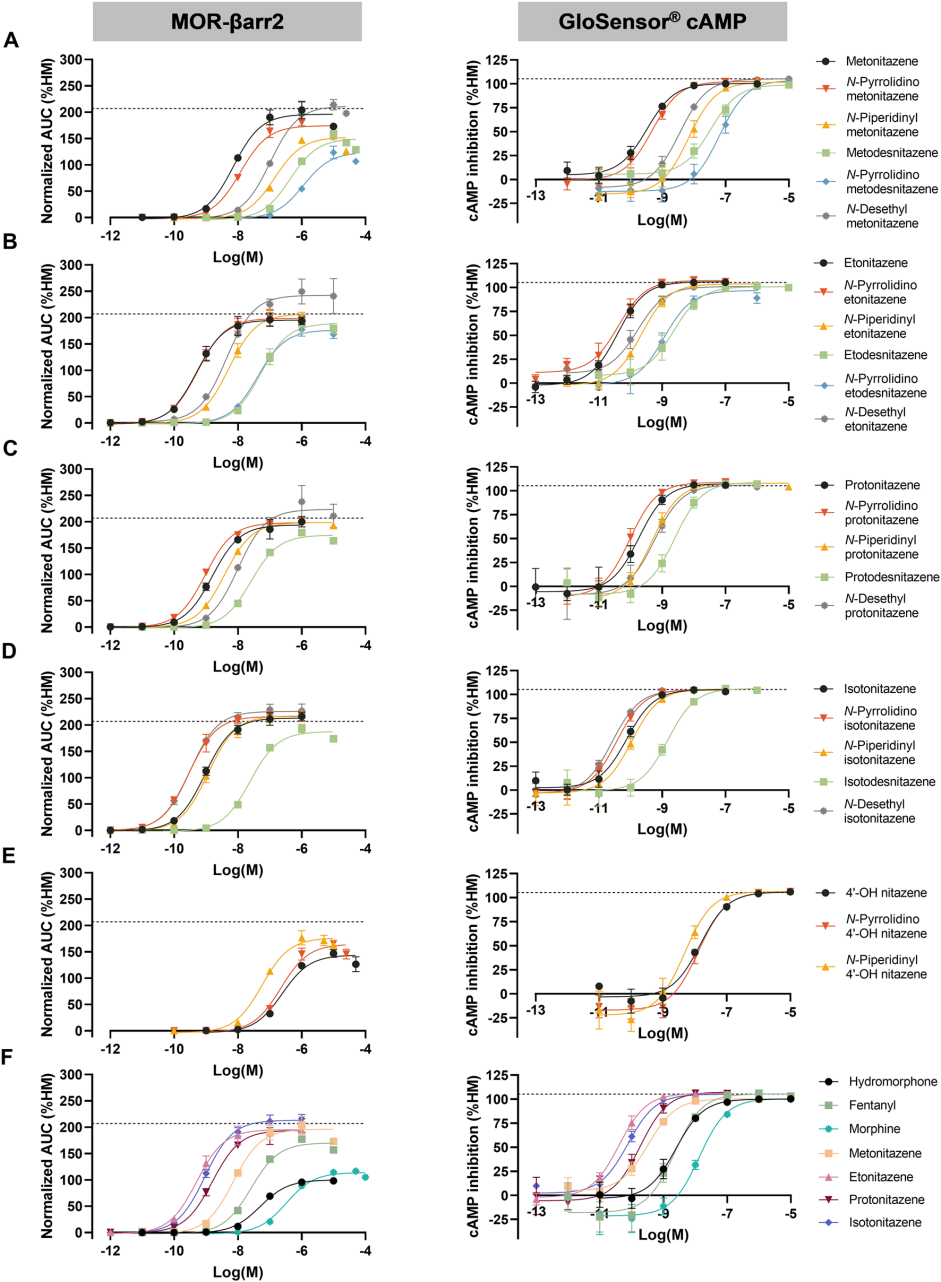
μ-Opioid receptor (MOR) activation profiles (MOR-βarr2 and GloSensor^®^ cAMP assays) for analogues of metonitazene (**A**), etonitazene (**B**), protonitazene (**C**), isotonitazene (**D**), and 4′-OH nitazene (**E**). Comparator drugs and reference nitazenes are shown in **F**. Data are presented as mean receptor activation ± standard error of the mean (SEM) (*n* ≥ 3) and are normalized to the *E*_max_ of hydromorphone (HM) (= 100%). The dotted line represents the maximum MOR activation produced by DAMGO. AUC, area under the curve. Note the differences in scales of the axes (color figure online)

**Fig. 4:**
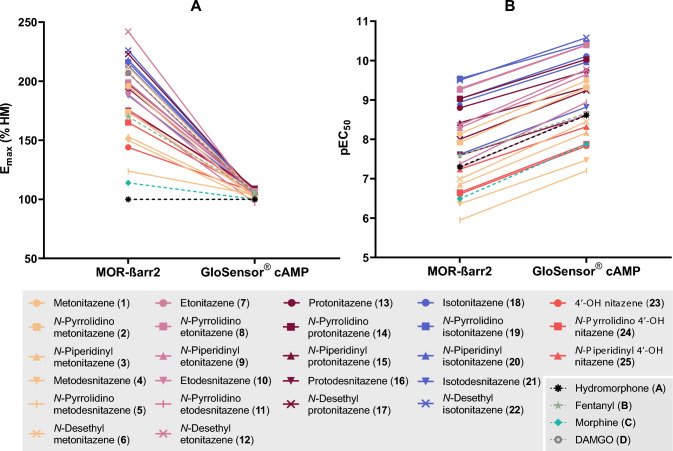
Comparison of obtained efficacies (*E*_max_, relative to hydromorphone (HM)) (**A**) and potencies (pEC_50_) (**B**) of the evaluated compounds (**1**–**25**, **A**–**D**) in the MOR-βarr2 and the GloSensor^®^ cAMP assays (color figure online)

Two in vitro assays (MOR-βarr2 and GloSensor^®^ cAMP) were employed to evaluate the MOR activation potential of a set of 25 differentially substituted 2-benzylbenzimidazole opioids and 4 reference opioids. All compounds were active at MOR and full concentration–response curves were obtained for every compound (Fig. 3), except for DAMGO (**D**). None of the compounds exhibited aspecific signals (i.e., independent of MOR activation) in the GloSensor^®^ cAMP control experiments, except for *N*-pyrrolidino etodesnitazene (**11**) at the highest tested concentration (Fig. S3). Consequently, the data points for *N*-pyrrolidino etodesnitazene (**11**) corresponding to this concentration were excluded from the GloSensor^®^ cAMP dataset. The obtained functional parameters (potency and efficacy relative to hydromorphone (HM)) are summarized in Table 1. Efficacies relative to DAMGO (**D**) can be found in Table S4. All nitazenes were full agonists compared to hydromorphone (**A**). In the GloSensor^®^ cAMP assay, the efficacies converged around 100% (Fig. 4), while the *E*_max_ values ranged between 124 and 242% in the MOR-βarr2 assay. The efficacies of most of the evaluated nitazenes did not exceed that of DAMGO (*E*_max_ = 207% compared to HM) in this assay. The exceptions were *N*-desethyl etonitazene (**12**) (*E*_max_ = 242%) and *N*-desethyl isotonitazene (**22**) (*E*_max_ = 226%), which displayed higher efficacies than DAMGO (**D**) in the employed assay. All compounds showed higher absolute potencies (i.e., lower EC_50_ or higher pEC_50_ values) in the GloSensor^®^ cAMP assay (EC_50_ range: 0.0263–63.9 nM) compared to the MOR-βarr2 assay (EC_50_ range: 0.288–1124 nM) (Fig. 4). However, the relative rank order of potency was largely consistent across assays (Fig. 4). Etonitazene (**7**), *N*-pyrrolidino etonitazene (**8**), *N*-pyrrolidino isotonitazene (**19**), and *N*-desethyl isotonitazene (**22**) were consistently among the most potent analogues. In both assays, the potencies of these analogues exceeded that of morphine (**C**) and fentanyl (**B**) > 320 and > 45 times, respectively. *N*-Pyrrolidino metodesnitazene (**5**) was the least potent analogue in both assays (~ 3 and ~ 30 times less potent than morphine (**C**) and fentanyl (**B**), respectively). This compound was also the least efficacious analogue in the MOR-βarr2 assay (*E*_max_ = 124%). A visual ‘potency scale’ of the different compounds relative to fentanyl is provided in Fig. S5.

**Table 1:** Summary of potency (EC_50_ and pEC_50_) and efficacy (*E*_max_, relative to hydromorphone (HM)) measures for all nitazenes (**1**–**25**) and comparator drugs (**A**–**D**), as obtained in the MOR-βarr2 recruitment and GloSensor^®^ cAMP assays (*n* ≥ 3). For every nitazene, the table displays the substitutions of the 2-benzylbenzimidazole core structure as shown in Fig. 1. 95% confidence intervals (CI) are shown between parentheses

	R_1_	R_2_	R_3_	MOR–βarr2			GloSensor^®^ cAMP		
				EC_50_ (nM)	pEC_50_	*E*_max_ (% HM)	EC_50_ (nM)	pEC_50_	*E*_max_ (% HM)
**1**. Metonitazene^a,b^	–OCH_3_	–NO_2_	–N(C_2_H_5_)_2_	7.19 (4.56–11.2)	8.14 (7.95–8.34)	196 (183–210)	0.328 (0.201–0.535)	9.49 (9.27–9.70)	100 (94.9–106)
**2**. *N*-Pyrrolidino metonitazene^a,c^	–OCH_3_	–NO_2_	–N(C_4_H_8_)	12.0 (8.29–17.4)	7.92 (7.76–8.08)	174 (164–185)	0.475 (0.284–0.770)	9.32 (9.11–9.55)	103 (96.4–109)
**3**. *N*-Piperidinyl metonitazene^d^	–OCH_3_	–NO_2_	–N(C_5_H_10_)	141 (92.4–216)	6.85 (6.66–7.04)	153 (142–163)	6.78 (4.22–10.6)	8.17 (7.97–8.37)	102 (94.7–109)
**4**. Metodesnitazene^a,b^	–OCH_3_	–H	–N(C_2_H_5_)_2_	433 (258–715)	6.36 (6.15–6.59)	150 (138–162)	33.7 (16.2–69.1)	7.47 (7.16–7.79)	99.1 (89.4–109)
**5**. *N*-Pyrrolidino metodesnitazene^d^	–OCH_3_	–H	–N(C_4_H_8_)	1124 (638–1967)	5.95 (5.71–6.20)	124 (110–139)	63.9 (31.3–123)	7.20 (6.91–7.51)	104 (91.1–117)
**6**. *N*-Desethyl metonitazene^d^	–OCH_3_	–NO_2_	–NH(C_2_H_5_)	103 (68.3–155)	6.99 (6.81–7.17)	212 (198–225)	3.51 (2.22–5.52)	8.45 (8.26–8.65)	105 (98.8–112)
**7**. Etonitazene^a,b^	–OC_2_H_5_	–NO_2_	–N(C_2_H_5_)_2_	0.509 (0.321–0.796)	9.29 (9.10–9.49)	195 (184–207)	0.0398 (0.0272–0.0579)	10.4 (10.2–10.6)	106 (101–111)
**8**. *N*-Pyrrolidino etonitazene^a,b^	–OC_2_H_5_	–NO_2_	–N(C_4_H_8_)	0.548 (0.376–0.791)	9.26 (9.10–9.43)	199 (189–209)	0.0409 (0.0196–0.0826)	10.4 (10.1–10.7)	107 (99.0–116)
**9**. *N*-Piperidinyl etonitazene^a,b^	–OC_2_H_5_	–NO_2_	–N(C_5_H_10_)	5.13 (3.50–7.40)	8.29 (8.13–8.46)	207 (194–220)	0.222 (0.157–0.319)	9.65 (9.50–9.80)	104 (99.4–108)
**10**. Etodesnitazene^a,b,c^	–OC_2_H_5_	–H	–N(C_2_H_5_)_2_	51.9 (32.3–81.6)	7.29 (7.09–7.49)	189 (174–204)	2.32 (1.03–5.51)	8.64 (8.26–8.99)	101 (91.9–110)
**11**. *N*-Pyrrolidino etodesnitazene^d^	–OC_2_H_5_	–H	–N(C_4_H_8_)	41.4 (29.0–58.6)	7.38 (7.23–7.54)	176 (167–186)	1.16 (0.436–3.15)	8.94 (8.50–9.36)	96.9 (84.5–110)
**12**. *N*-Desethyl etonitazene^a,c^	–OC_2_H_5_	–NO_2_	–NH(C_2_H_5_)	4.36 (2.30–8.02)	8.36 (8.10–8.64)	242 (224–261)	0.172 (0.0926–0.338)	9.77 (9.47–10.0)	101 (94.5–108)
**13**. Protonitazene^a,b^	–OC_3_H_7_	–NO_2_	–N(C_2_H_5_)_2_	1.57 (1.05–2.41)	8.80 (8.62–8.98)	194 (183–206)	0.185 (0.0828–0.434)	9.73 (9.36–10.1)	107 (94.2–121)
**14**. *N*-Pyrrolidino protonitazene^a,c^	–OC_3_H_7_	–NO_2_	–N(C_4_H_8_)	0.942 (0.811–1.09)	9.03 (8.96–9.09)	198 (194–202)	0.0939 (0.0531–0.167)	10.0 (9.78–10.3)	109 (99.8–118)
**15**. *N*-Piperidinyl protonitazene^d^	–OC_3_H_7_	–NO_2_	–N(C_5_H_10_)	3.78 (3.15–4.51)	8.42 (8.35–8.50)	199 (194–203)	0.480 (0.270–0.833)	9.32 (9.08–9.57)	108 (100–116)
**16**. Protodesnitazene^d^	–OC_3_H_7_	–H	–N(C_2_H_5_)_2_	25.6 (19.9–33.1)	7.59 (7.48–7.70)	175 (168–182)	2.50 (1.37–4.67)	8.60 (8.33–8.86)	109 (98.3–120)
**17**. *N*-Desethyl protonitazene^d^	–OC_3_H_7_	–NO_2_	–NH(C_2_H_5_)	10.1 (5.45–18.8)	8.00 (7.73–8.26)	223 (204–243)	0.580 (0.259–1.24)	9.24 (8.91–9.59)	106 (94.6–118)
**18**. Isotonitazene^a,b^	–OCH(CH_3_)_2_	–NO_2_	–N(C_2_H_5_)_2_	0.923 (0.696–1.23)	9.04 (8.91–9.16)	213 (205–222)	0.0769 (0.0488–0.120)	10.1 (9.92–10.3)	105 (98.8–112)
**19**. *N*-Pyrrolidino isotonitazene	–OCH(CH_3_)_2_	–NO_2_	–N(C_4_H_8_)	0.288 (0.202–0.414)	9.54 (9.38–9.70)	216 (207–225)	0.0366 (0.0228–0.0581)	10.4 (10.2–10.6)	105 (99.2–111)
**20**. *N*-Piperidinyl isotonitazene	–OCH(CH_3_)_2_	–NO_2_	–N(C_5_H_10_)	1.16 (0.840–1.61)	8.94 (8.79–9.08)	218 (208–228)	0.110 (0.0610–0.198)	9.96 (9.70–10.2)	106 (97.0–115)
**21**. Isotodesnitazene	–OCH(CH_3_)_2_	–H	–N(C_2_H_5_)_2_	24.0 (17.9–32.4)	7.62 (7.49–7.75)	188 (179–196)	1.53 (0.829–2.91)	8.82 (8.54–9.08)	106 (96.8–116)
**22**. *N*-Desethyl isotonitazene^a,b,c^	–OCH(CH_3_)_2_	–NO_2_	–NH(C_2_H_5_)	0.317 (0.225–0.447)	9.50 (9.35–9.65)	226 (217–235)	0.0263 (0.0182–0.0384)	10.6 (10.4–10.7)	105 (100–109)
**23**. 4′-OH nitazene	–OH	–NO_2_	–N(C_2_H_5_)_2_	243 (150–395)	6.62 (6.40–6.82)	144 (132–156)	14.9 (8.49–27.3)	7.83 (7.56–8.07)	106 (96.7–115)
**24**. *N*-Pyrrolidino 4′-OH nitazene^d^	–OH	–NO_2_	–N(C_4_H_8_)	222 (151–328)	6.65 (6.48–6.82)	165 (154–176)	13.1 (7.56–23.4)	7.88 (7.63–8.12)	106 (96.0–116)
**25**. *N*-Piperidinyl 4′-OH nitazene^d^	–OH	–NO_2_	–N(C_5_H_10_)	56.9 (38.3–83.4)	7.25 (7.08–7.42)	176 (166–187)	4.75 (2.17–9.95)	8.32 (8.00–8.66)	107 (94.3–119)
**A**. Hydromorphone				49.7 (39.8–61.7)	7.30 (7.21–7.40)	100 (96.3–104)	2.45 (1.26–4.91)	8.61 (8.31–8.90)	100 (91.8–109)
**B**. Fentanyl				25.7 (19.2–34.6)	7.59 (7.46–7.72)	170 (163–178)	2.20 (1.06–4.82)	8.66 (8.32–8.98)	106 (93.6–118)
**C**. Morphine				327 (239–448)	6.49 (6.35–6.62)	114 (109–119)	13.2 (6.12–29.4)	7.88 (7.53–8.21)	100 (87.4–114)
**D**. DAMGO				–	–	207 (203–212)	–	–	105 (94.5–117)

^a^Analogues that have been identified on the recreational drug market in the U.S. (cfr. Fig. 2)

^b^Analogues placed under Schedule I control by the DEA

^c^Analogues for which forensic cases are reported in the present study

^d^Previously uncharacterized nitazenes (9 in total)

### Toxicology results, prevalence, and geographical distribution of etodesnitazene, *N*-desethyl etonitazene, *N*-desethyl isotonitazene, *N*-pyrrolidino metonitazene, and *N*-pyrrolidino protonitazene

A total of 85 cases (84 postmortem and one DUID) from North America and the U.K. were investigated (Table S6). The average age was 41 ± 14 years (median 40 years) and ranged from 19 to 75 years. Etodesnitazene was confirmed in 26 individual postmortem cases collected between May 2020 and May 2023. Based on quantitative data from 15 cases, the concentration range of etodesnitazene in blood was 0.1–120 ng/mL. *N*-Desethyl isotonitazene, a metabolite of isotonitazene (Krotulski et al. 2020) but also known to be marketed and sold in its own right, was detected in 15 fatalities and one DUID investigation (December 2022 to November 2023). Quantitative results were obtained for the DUID and 8 postmortem cases (concentration range: 0.82–8.3 ng/mL). Note that cases where *N*-desethyl isotonitazene was found alongside isotonitazene were excluded. *N*-Desethyl etonitazene was detected in one case in the absence of other opioids (**case 79** from July 2023). However, quantitative analysis of *N*-desethyl etonitazene was not performed. *N*-Desethyl protonitazene was mainly found in combination with protonitazene, in which case it was considered a metabolite. However, *N*-desethyl protonitazene was found in the absence of protonitazene in two cases (Table S6-2/3, **case 45** from June 2023 and **case 73** from March 2023). In one of these (**case 45**), it was quantified at a concentration of 0.1 ng/mL. Nevertheless, *N*-desethyl protonitazene was also considered as a metabolite rather than a standalone drug in both cases since, in contrast to *N*-desethyl isotonitazene and *N*-desethyl etonitazene, it is not yet known to be sold as a drug on its own. The presence of *N*-pyrrolidino protonitazene was confirmed in 39 postmortem investigations collected between December 2022 and September 2023. Interestingly, *N*-pyrrolidino metonitazene was concurrently detected in 13 of these cases, and was identified without *N*-pyrrolidino protonitazene in only two cases (**case 62** from August 2023 and **case 67** from June 2023). *N*-Pyrrolidino protonitazene and *N*-pyrrolidino metonitazene could be quantified in 26 and 11 cases, with blood concentration ranges of 0.3–55 and 0.2–26 ng/mL, respectively. The aggregated quantitative results for each analogue are shown in Table 2. The majority of cases originated from North America, with 64 cases from the U.S. and 10 cases from Canada. Eleven cases were from the U.K., where *N*-desethyl isotonitazene was detected in 5 cases and *N*-pyrrolidino protonitazene in 6 cases. *N*-pyrrolidino metonitazene was also concurrently detected in one of these *N*-pyrrolidino protonitazene cases (**case 64**). The detailed toxicology results, demographics (e.g., age, sex, city, and state) and short history of all evaluated cases are included in Table S6-1/2/3.

**Table 2:** Overview of the mean, median, and range of blood concentrations for etodesnitazene, *N*-desethyl isotonitazene, *N*-pyrrolidino metonitazene, and *N*-pyrrolidino protonitazene identified in individual forensic cases

Compound of interest	*N* Qualitative	*N* Quantitative	Mean (±SD) (ng/mL)	Median (ng/mL)	Range (ng/mL)
Etodesnitazene	26	15	25 ± 36	4	0.1–120
*N*-Pyrrolidino protonitazene	39	26	8 ± 17	1.2	0.3–55
*N*-Pyrrolidino metonitazene	15	11	3 ± 7	0.47	0.2–26
*N*-Desethyl isotonitazene	16	9	4 ± 2	3.4	0.82–8.3

*N* = number of cases

**Fig. 5:**
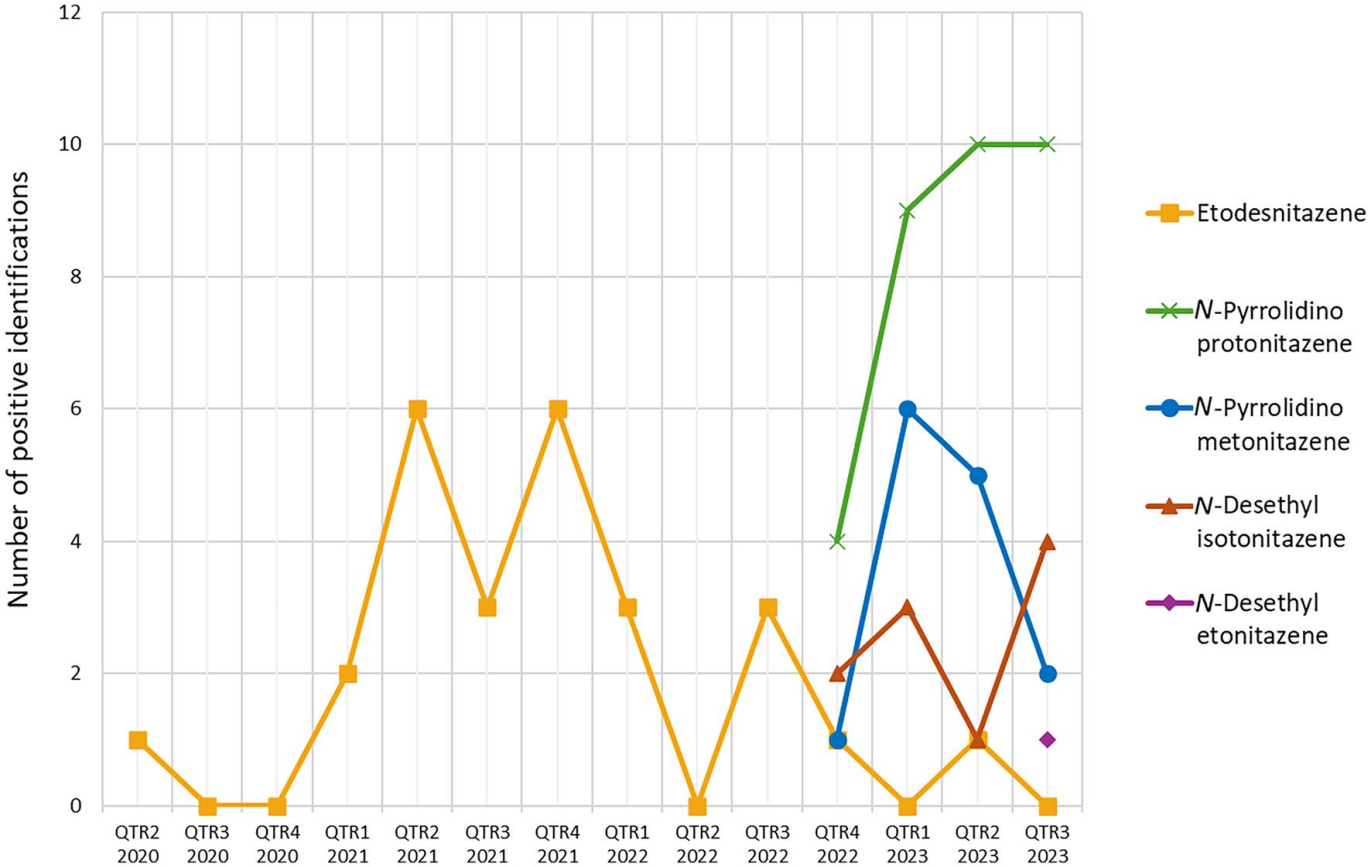
Prevalence of etodesnitazene (yellow squares), *N*-pyrrolidino protonitazene (green crosses), *N*-pyrrolidino metonitazene (blue circles), *N*-desethyl isotonitazene (red triangles), and *N*-desethyl etonitazene (purple diamond) between Q2 2020 and Q3 2023 in North America, according to the results of comprehensive toxicological testing by the CFSRE, at the time of data compilation (Q1 2024). Unless noted in Table S6-1/2/3, dates represent the date of sample collection. Etodesnitazene emerged in Q2 2020 and reached peak prevalence in 2021. *N*-Pyrrolidino protonitazene, *N*-pyrrolidino metonitazene, and *N*-desethyl isotonitazene all emerged in Q4 2022, while *N*-desethyl etonitazene emerged in Q3 2023. The 11 reported cases from the U.K. (5 involving *N*-pyrrolidino protonitazene, from Q2 to Q3 2023, 5 involving *N*-desethyl isotonitazene, from Q3 to Q4 2023, and 1 involving both *N*-pyrrolidino protonitazene and *N*-pyrrolidino metonitazene, from Q3 2023) are not included in the figure. Note that some cases were positive for more than one of the listed nitazenes (color figure online)

Figure 5 displays the prevalence over time of the North American cases of etodesnitazene, *N*-desethyl etonitazene, *N*-desethyl isotonitazene, *N*-pyrrolidino metonitazene, and *N*-pyrrolidino protonitazene. These data suggest that etodesnitazene reached peak positivity in 2021 with 17 positive cases that year, and its positivity decreased in 2022 (7 cases) and 2023 (Q1-Q3, 1 case). Notably, Q4 2022 marked the emergence of *N*-desethyl isotonitazene, *N*-pyrrolidino metonitazene, and *N*-pyrrolidino protonitazene, with their respective positivities further increasing through the first quarter of 2023. Since then, the prevalence of *N*-pyrrolidino protonitazene has largely remained stable, while the positivities for *N*-desethyl isotonitazene and *N*-pyrrolidino metonitazene have fluctuated and decreased, respectively. *N*-Desethyl etonitazene was identified for the first time in Q3 2023 (1 case).

**Fig. 6:**
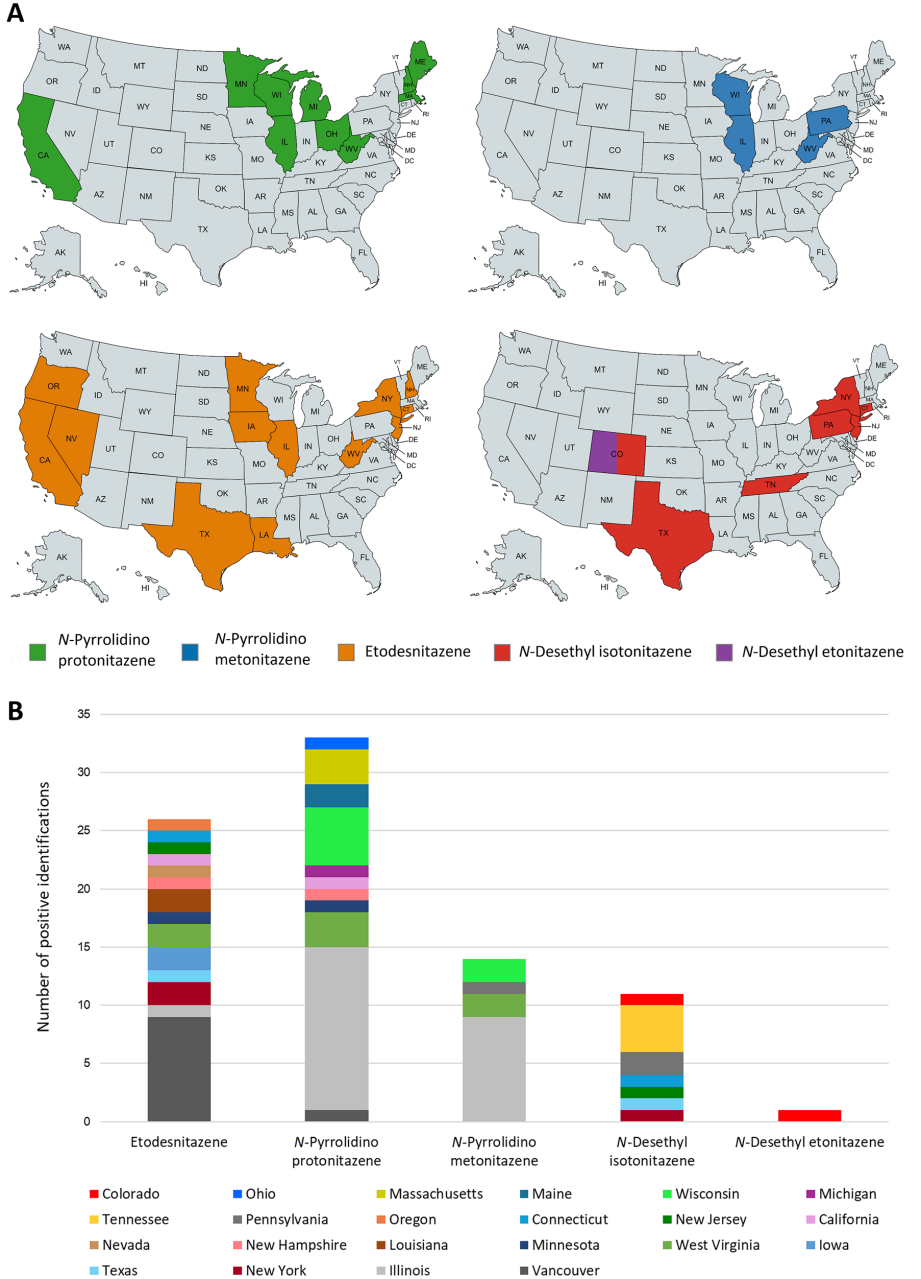
Geographical distribution of etodesnitazene, *N*-pyrrolidino protonitazene, *N*-pyrrolidino metonitazene, *N*-desethyl etonitazene, and *N*-desethyl isotonitazene cases in the U.S. (9 cases for etodesnitazene and 1 case for *N*-pyrrolidino protonitazene from Vancouver, Canada, are also included in the bar chart in **B**). The U.S. maps shown in **A** were created with mapchart.net (color figure online)

The geographical distribution of the 5 nitazenes in North America is depicted in Fig. 6. The 26 etodesnitazene cases were from a total of 13 states. Notably, 9 of 26 cases were from Vancouver, Canada. The 32 *N*-pyrrolidino protonitazene and 14 *N*-pyrrolidino metonitazene cases from the U.S. came from 10 and 4 different states, respectively. Notably, a substantial number of these cases originated from Illinois (14 of 32 *N*-pyrrolidino protonitazene and 9 of 14 *N*-pyrrolidino metonitazene cases). Furthermore, all 9 *N*-pyrrolidino metonitazene cases that came from Illinois were instances where this nitazene was co-detected with *N*-pyrrolidino protonitazene. For *N*-desethyl isotonitazene, the 11 U.S. cases were from 7 different states. The single *N*-desethyl etonitazene case was from Colorado.

With the exception of one etodesnitazene case (**case 6**), all of the evaluated cases (*n* = 85) were polydrug intoxications. Fentanyl and xylazine were identified in 56% (*n* = 48) and 31% of the cases (*n* = 26), respectively. Note that, with the exception of two *N*-desethyl isotonitazene cases (**cases 70** and **83**), xylazine was always found together with fentanyl. Amphetamine and methamphetamine were both identified in 24% (*n* = 20) of the cases and a diverse panel of benzodiazepines was also detected in 64% of the cases (*n* = 54). Other nitazenes (not classified as metabolites) were also identified, including metonitazene (*n* = 27), isotonitazene (*n* = 6), protonitazene (*n* = 13), and *N*-pyrrolidino etonitazene (*n* = 1). Other detected NSOs were predominantly fentanyl analogues and included *para*-fluorofentanyl (*n* = 12), acetyl fentanyl (*n* = 11), acrylfentanyl (*n* = 1), and butyrylfentanyl (*n* = 1). Additional non-fentanyl NSOs identified included mitragynine (*n* = 7) and brorphine (*n* = 1).

In 6 cases (**cases 2**, **6**, **8**, **9**, **10**, and **26**), etodesnitazene was detected in the absence of other opioid agonists. In two of these cases, etodesnitazene was quantified at concentrations of 1.8 ng/mL (**case 9**) and 69 ng/mL (**case 10**). *N*-Pyrrolidino protonitazene was detected without other opioid agonists in 5 cases (**cases 29**, **44**, **50**, **61**, and **65**) and was quantified in 4 of these cases at concentrations of 1.1 ng/mL (**case 29**), 0.3 ng/mL (**case 44**), 55 ng/mL (**case 50**), and 15 ng/mL (**case 61**). In the two cases where *N*-pyrrolidino metonitazene was not co-detected with *N*-pyrrolidino protonitazene (**cases 62** and **67**), *N*-pyrrolidino metonitazene was detected and quantified without other opioid agonists in one case at a concentration of 26 ng/mL (**case 67**). *N*-Desethyl isotonitazene was identified without other opioid agonists in 4 cases (**cases 68**, **70**, **80**, and **82**) and could be quantified in one case (**case 68**) at a concentration of 0.82 ng/mL. In the one case where *N*-desethyl etonitazene was detected (**case 79**), it was found without other opioids.

## Discussion

NSOs with a 2-benzylbenzimidazole core (‘nitazenes’) continue to emerge on recreational drug markets. *N*-Pyrrolidino metonitazene, *N*-pyrrolidino protonitazene, and *N*-desethyl etonitazene are among the most recent nitazenes identified in the U.S. (Krotulski et al. 2023). Although great strides have been made in the evaluation of nitazene structure–activity relationships (SAR) (Ujváry et al. 2021; Vandeputte et al. 2021, 2022b, e, 2023; De Luca et al. 2022; Kanamori et al. 2023; Walton et al. 2023; Malcolm et al. 2023; Glatfelter et al. 2023; Kozell et al. 2024; Tsai et al. 2024), larger side-by-side comparisons of the effect of different systematic modifications to the nitazene core structure on MOR activity are rather scarce. Specifically, our understanding of SAR for ‘ring’ substituted analogues (i.e., *N*-pyrrolidino and *N*-piperidinyl analogues) remains limited (Ujváry et al. 2021; Vandeputte et al. 2022b, e; Kozell et al. 2024; Tsai et al. 2024). For example, similar to *N*-pyrrolidino etonitazene (Vandeputte et al. 2022b), *N*-pyrrolidino metonitazene and *N*-pyrrolidino protonitazene were not included in the early CIBA studies, and can thus be considered truly ‘new’ synthetic opioids. To further unravel nitazene SAR, we performed in vitro pharmacological characterization of a panel of 25 differentially substituted nitazenes, including 9 previously uncharacterized analogues, focusing on three distinct positions of the 2-benzylbenzimidazole core structure (R_1–3_ in Fig. 1 and Table 1). In addition, complementing the in vitro pharmacological findings and demonstrating the danger posed by these substances to the users, we report the first identification and toxicological analysis of etodesnitazene, *N*-desethyl etonitazene, *N*-desethyl isotonitazene, *N*-pyrrolidino metonitazene, and *N*-pyrrolidino protonitazene in forensic cases from North America as well as the identification of *N*-pyrrolidino metonitazene, *N*-pyrrolidino protonitazene, and *N*-desethyl isotonitazene in cases from the U.K. Hence, in line with earlier recommendations that the opioid crisis should be dealt with in a multidisciplinary manner (Baumann et al. 2018; Morrow et al. 2019), and not solely by, e.g., isolated case reports or pharmacology-only studies, this study aimed at bridging these two areas. The latter is also particularly relevant for the different stakeholders involved, as observations in forensic casework can be supported by pharmacological data. This is a prerequisite when considering, e.g., both scheduling efforts and harm reduction strategies.

### In vitro SAR determination

In this study, two distinct cell-based assays were used for the functional characterization of a series of nitazenes at MOR: a β-arrestin 2 recruitment assay, which monitors receptor-proximal (upstream) activation of the G protein-independent pathway, and a GloSensor^®^ cAMP assay, which monitors the inhibition of forskolin-stimulated cAMP accumulation downstream of G_αi_ protein activation. While measuring MOR activation at different levels (upstream and further downstream) in a common cellular background (HEK 293T cells) can be considered a strength of this study, it is important to consider some inherent differences in the characteristics of the assays that were deployed (Zhang and Xie 2012; Vandeputte et al. 2022d). For example, the comparatively higher level of signal amplification in the cAMP assay, as well as the modification of MOR and co-expression of GRK2 in the βarr2 recruitment assay, are factors to be considered. Evaluation of these assay-specific parameters was beyond the scope of this study, which aimed at determining nitazene SAR using assays that have been extensively used before, by us and others (Vandeputte et al. 2022d). In this context, it is important to note that all efficacies in the cAMP assay converged around ~ 100% compared to hydromorphone, hampering a comparative assessment of the efficacies of the evaluated compounds. This ‘plateauing effect’ is a well-known characteristic of assays assessing downstream effects, such as the cAMP assay (Vandeputte et al. 2022d). The MOR-βarr2 assay, in contrast, has historically been demonstrated to yield a wide range of efficacies (Vandeputte et al. 2021, 2022c). For many of the compounds evaluated here, the *E*_max_ approached the efficacy of DAMGO. For some (isotonitazene (**18**), *N*-pyrrolidino isotonitazene (**19**), *N*-piperidinyl isotonitazene (**20**) and the evaluated *N*-desethyl analogues (**6**, **12**, **17** and **22**)), the efficacy even surpassed that of DAMGO, although in most instances the confidence intervals were still overlapping with that of DAMGO. This is in line with observations by others, some of whom have used the term MOR ‘superagonism’ to refer to MOR activation induced by several nitazenes (Malcolm et al. 2023; Tsai et al. 2024). While absolute potencies differed in both our assays, the inclusion of multiple comparator opioids in this study (**A**–**C**) allowed a comparative assessment of potencies (Vandeputte et al. 2022c) that was highly similar between both assays. This strengthens the observed nitazene SAR trends, which are discussed below. Importantly, while there are clear limitations to the translatability of in vitro studies to the in vivo situation (e.g., because bioavailability, blood–brain barrier penetration, and formation of active metabolites are not taken into account), prior studies have shown that in vitro functional assays at MOR (including the MOR-βarr2 assay employed here) can serve as a tool for predicting in vivo potency trends of NSOs (including nitazenes) in rodents (Vandeputte et al. 2022b, 2023; Glatfelter et al. 2023). Therefore, the proposed in vitro SAR trends, indicating a (very) high MOR activating potential of many of the investigated compounds, may be cautiously extrapolated to the in vivo setting (Gillis et al. 2020; Tsai et al. 2024). Related to the latter, as elaborated below, the inclusion of forensic case series (although typically confounded by the co-presence of other drugs, including opioids) allowed to corroborate the in vitro findings, by demonstrating that these substances are found at low concentrations in fatal intoxications.

Structural modifications of the hitherto identified nitazenes (Fig. 2) are confined to three specific regions of the 2-benzylbenzimidazole template (R_1–3_ in Fig. 1 and Table 1) (Glatfelter et al. 2023). Considering the *para*-benzyl position (R_1_), prior research has shown that the length of the *para*-alkoxy side chain dictates the impact on potency, with intermediate chain lengths (i.e., ethyl and isopropyl) being most optimal for MOR activation. Shorter (i.e., methyl) or longer (i.e., propyl and butyl) tails lead to less potent analogues (Vandeputte et al. 2021; Kanamori et al. 2023; Glatfelter et al. 2023; Kozell et al. 2024). Substitution of the alkoxy chain by a halogen atom or free phenol greatly decreases the MOR activity to a level more comparable to that of morphine (Hunger et al. 1960b; Vandeputte et al. 2021; Kozell et al. 2024). The in vitro potency trends of all reference nitazenes included in this study are consistent with the abovementioned findings.

Elimination of the nitro group at the 5-position of the benzimidazole ring (R_2_) generates so-called ‘desnitro’ analogues or ‘desnitazenes’. Previous in vitro and in vivo data showed that removal of the 5-nitro group leads to a significant reduction in potency (Hunger et al. 1960a; Vandeputte et al. 2021; Kozell et al. 2024). In line with those studies, our findings for metodesnitazene, etodesnitazene, and isotodesnitazene confirm that absence of the nitro group reduces the potency approximately 10–100-fold. The newly characterized protodesnitazene follows this trend, displaying a > 13-fold lower potency than protonitazene in both assays.

Different modifications of the R_3_ position of the nitazene core structure are possible. The two ethyl groups of the common *N*,*N*-diethyl amine group can be substituted for a pyrrolidine or piperidine moiety, leading to the formation of ‘ring’ nitazenes. Previous in vitro studies (Vandeputte et al. 2022b, e; Tsai et al. 2024) showed that both *N*-pyrrolidino and *N*-piperidinyl substitutions of the amine side chain are well-tolerated in terms of MOR activation, with *N*-pyrrolidino etonitazene being equally potent and *N*-piperidinyl etonitazene being somewhat less potent than etonitazene. Furthermore, Kozell et al. (2024) observed similar trends across a larger panel of ‘ring’ nitazenes. In that study, *N*-piperidinyl etonitazene and *N*-piperidinyl isotonitazene displayed a slightly lower or similar potency compared to etonitazene and isotonitazene, respectively, and different *N*-pyrrolidino analogues (*N*-pyrrolidino etonitazene, *N*-pyrrolidino isotonitazene, *N*-pyrrolidino protonitazene, and *N*-pyrrolidino metonitazene) displayed a similar or slightly higher potency than their corresponding *N*,*N*-diethyl amine equivalents. By contrast, *N*-pyrrolidino metonitazene was about two times less potent than metonitazene (Kozell et al. 2024). These findings are broadly in line with our results, and further building on ‘ring’ nitazene SAR, we show that these modifications generally yield highly active drugs, with a trend of *N*-pyrrolidino analogues being more potent than the corresponding *N*-piperidinyl analogues. A possible exception to this trend was observed for analogues of 4′-OH nitazene: here, the *N*-piperidinyl analogue displayed a similar or slightly higher potency than the corresponding *N*-pyrrolidino analogue. Furthermore, with the exception of *N*-piperidinyl 4′-OH nitazene, piperidine analogues are generally less potent than the corresponding *N*,*N*-diethyl amine analogues. The decrease in potency was most pronounced (i.e., 20x) for *N*-piperidinyl metonitazene, the analogue with the shortest alkoxy side chain. Upon lengthening the alkoxy tail, the potency shift appeared to become progressively smaller, with *N*-piperidinyl etonitazene being > 5 times less potent and *N*-piperidinyl protonitazene being equipotent or slightly less potent than the corresponding *N*,*N*-diethyl amine equivalents. For *N*-piperidinyl isotonitazene, no potency shift compared to isotonitazene was observed with the employed assays. In contrast, pyrrolidino analogues were generally about equipotent as their *N*,*N*-diethyl amine counterparts.

4′-OH nitazene is a universal metabolite of all nitazenes with alkoxy chain modifications only (Taoussi et al. 2024; Kanamori et al. 2024; Walton et al. 2021). Similarly, based on recent findings for *N*-piperidinyl etonitazene where the *N*-piperidinyl 4′-OH nitazene metabolite was identified in authentic urine samples from two patients that took this compound (Vandeputte et al. 2022e; Berardinelli et al. 2024), it can be hypothesized that *N*-pyrrolidino 4′-OH nitazene and *N*-piperidinyl 4′-OH nitazene are common *O*-dealkylated metabolites of the herein evaluated ‘ring’ nitazenes. In support of this hypothesis, *N*-pyrrolidino 4′-OH nitazene was detected in the blood samples of two *N*-pyrrolidino protonitazene cases (**cases 52** and **61**) included in Table S6-2. While a quantitative assessment of this metabolite was not performed, its detection in only 2/41 cases involving *N*-pyrrolidino metonitazene and/or *N*-pyrrolidino protonitazene might indicate its presence at considerably lower concentrations than the parent drugs in blood, as also seen for 4′-OH nitazene (Walton et al. 2023). Combined with in vitro findings, which suggest that *N*-pyrrolidino 4′-OH nitazene and *N*-piperidinyl 4′-OH nitazene are generally less potent than the evaluated ‘ring’ nitazenes, it is likely that any potential impact of the activity of these metabolites on the overall in vivo effects of the parent ‘ring’ nitazene is limited. Conversely, in the case of *N*-piperidinyl metonitazene, the expected *N*-piperidinyl 4′-OH nitazene metabolite may be equally or slightly more active than the parent compound itself. However, dedicated studies on the in vivo human metabolism of ‘ring’ nitazenes are warranted (Berardinelli et al. 2024).

*N*-Pyrrolidino metodesnitazene and *N*-pyrrolidino etodesnitazene were included in this study to assess the effect of combining a pyrrolidine function (known to be well-tolerated in terms of MOR activation potential, cfr. supra) with removal of the 5-nitro group, known to negatively impact opioid activity. The opioid activity of *N*-pyrrolidino etodesnitazene was readily reported in 1960 by means of a mouse tail-flick assay (Hunger et al. 1960a), which revealed an antinociceptive potency 20 times greater than that of morphine. In agreement with these findings, our in vitro data show that *N*-pyrrolidino etodesnitazene is at least 8 times more potent than morphine. As anticipated, *N*-pyrrolidino metodesnitazene had a pronounced lower potency (i.e., ~ 100×) than *N*-pyrrolidino metonitazene. In line with the equipotency observed for *N*-pyrrolidino metonitazene and metonitazene in our assays, the potencies of *N*-pyrrolidino metodesnitazene and metodesnitazene are of the same order of magnitude. Similar trends were observed for *N*-pyrrolidino etodesnitazene. Hence, the combined impact on in vitro MOR activity of the two structural alterations (‘ring’ modification + removal of the 5-nitro group) is roughly equivalent to the cumulative effect of each individual modification, with the substantial loss of potency being attributable to the removal of the 5-nitro group.

Removal of one ethyl moiety from the *N*,*N*-diethyl amine side chain (R_3_) yields *N*-desethyl nitazenes. Prior pharmacological assessments of *N*-desethyl etonitazene and *N*-desethyl isotonitazene showed that these analogues have important MOR activity (Vandeputte et al. 2021, 2023; Walton et al. 2023; Malcolm et al. 2023; Kozell et al. 2024; Tsai et al. 2024). Further exploring these SAR, our results from both MOR-βarr2 and GloSensor^®^ cAMP assays showed that the *N*-desethyl modification mostly results in a lower potency compared to the respective *N*,*N*-diethyl equivalents. Intriguingly, *N*-desethyl isotonitazene showed the opposite trend, with a ~ threefold increase in potency compared to isotonitazene in both assays. While this is in line with previous in vitro (Vandeputte et al. 2021, 2023; Kozell et al. 2024; Tsai et al. 2024) and in vivo (rat) findings (Walton et al. 2023; Vandeputte et al. 2023), Malcolm et al. (2023) did not observe this trend in in vitro and in vivo assays. This apparent discrepancy might be attributed to the use of different assays and/or experimental conditions.

*N*-Desethyl etonitazene and *N*-desethyl isotonitazene are metabolites of etonitazene and isotonitazene, respectively (Taoussi et al. 2024; Kanamori et al. 2024; Krotulski et al. 2020). As previously discussed for *N*-piperidinyl 4′-OH nitazene, the highly potent *N*-desethyl metabolites may theoretically contribute to the toxicity of the parent drug. Research (Walton et al. 2023) found that the highest concentration of *N*-desethyl isotonitazene in rats was at least 20 times lower than that of the parent molecule. Therefore, the in vivo relevance of *N*-desethyl metabolites may be limited, at least in rats. However, these analogues may pose significant health risks when used as standalone drugs. This is demonstrated by the 16 reported toxicological cases where *N*-desethyl isotonitazene was detected without isotonitazene, and one case where *N*-desethyl etonitazene was found without etonitazene. *N*-Desethyl isotonitazene is of particular concern, as it may combine high MOR binding affinity and functional potency (Walton et al. 2023; Vandeputte et al. 2023; Kozell et al. 2024) with prolonged respiratory depression in vivo (Malcolm et al. 2023). Given that, in protonitazene cases, *N*-desethyl protonitazene is typically only present to a minor extent, we speculate that the standalone presence of *N*-desethyl protonitazene in two cases from March 2023 and from June 2023 (Table S6-2/3, **cases 73** and **45**), in the absence of protonitazene, may indicate that also this metabolite may be available as such. However, confirmation of this scenario requires the detection of *N*-desethyl protonitazene without protonitazene in more toxicology cases and/or in drug material.

When considering the different modifications (R_1–3_) together, it is interesting to note that the ‘classic’ nitazenes differing in the alkoxy chain length (represented by the black curves in Fig. 3A–D) are generally the most potent (i.e., most left-shifted) of the respective panels. With the exception of *N*-pyrrolidino analogues, all other modifications systematically reduced the potency of the respective alkoxy chain variants (i.e., rightward shifts of the concentration–response curves). The isotonitazene scaffold is an interesting exception: only isotodesnitazene was substantially less potent than isotonitazene, and *N*-desethyl isotonitazene even somewhat exceeded the potency of isotonitazene in both assays. More research is needed to unravel the molecular basis of this watching compared to other nitazenes.

In terms of efficacies, most of the 9 previously uncharacterized analogues exhibit *E*_max_ values on par with those of known nitazenes in the MOR-βarr2 assay, often even surpassing that of fentanyl. Together with the high potencies, it can thus be expected that the intrinsic harm potential of the newly assessed nitazenes may be similar to that of other, previously identified nitazenes. As previously hypothesized by Malcolm et al. (2023), the ability of nitazenes to achieve high-signaling efficacy at MOR might stem from the induction of a unique and stable receptor conformational state that is able to robustly initiate both G protein activation and β-arrestin recruitment. In addition, it has been hypothesized that nitazenes have a long MOR residence time, maintaining the receptor in an active state for a longer period (Malcolm et al. 2023). In agreement with this, recent in silico studies confirm that nitazenes may have a MOR binding pose that is distinct from other opioids (De Luca et al. 2022; Chaturvedi et al. 2023). Future structural studies of MOR bound to 2-benzylbenzimidazoles may provide an explanation for the nitazene SAR trends observed by us and others.

### Forensic case series of recent nitazenes

Etodesnitazene, *N*-desethyl etonitazene, *N*-desethyl isotonitazene, *N*-pyrrolidino metonitazene, and *N*-pyrrolidino protonitazene were identified as toxicologically significant findings in various forensic cases originating from the U.S., Canada, and the U.K. between May 2020 and November 2023. The age distribution among individuals in the case series was wide (19–75 years). While not uncommon, it is interesting to note 4 cases (**cases 32**, **35**, **36**, and **72**) where individuals were aged over 66 years. Etodesnitazene, which was first identified in the U.S. in Q2 2020, peaked in positivity in 2021 and has decreased in prevalence since, possibly due to its relatively lower potency (the DEA published a notice of intent to schedule it in December 2021). The current data suggest that the prevalence of *N*-pyrrolidino protonitazene further increased and subsequently stabilized, while the positivity for *N*-desethyl isotonitazene has fluctuated since Q4 2022. In contrast, *N*-pyrrolidino metonitazene reached peak prevalence in Q1 2023 and has since declined in prevalence.

When examining the current geographical distribution of these 5 nitazenes, it is important to note that there is a sampling bias caused by coroners and medical examiners submitting to the CFSRE and NMS Labs. As a result, U.S. states from which both labs typically receive fewer samples, such as those in the Mountain West region, for example, will be underrepresented. When comparing the current geographical distribution of etodesnitazene, *N*-pyrrolidino protonitazene, and *N*-desethyl isotonitazene with that of other recent NSOs such as isotonitazene (Krotulski et al. 2020), brorphine (Krotulski et al. 2021a), metonitazene (Krotulski et al. 2021b), and (to a lesser extent) *N*-pyrrolidino etonitazene (Vandeputte et al. 2022b), it appears that their distribution across the U.S. is broader and more dispersed. However, *N*-pyrrolidino metonitazene and *N*-pyrrolidino protonitazene cases currently seem to be concentrated around the Midwestern U.S., mirroring the geographical distribution of isotonitazene and brorphine, for which the majority of cases initially originated from Illinois and other Midwestern states (Krotulski et al. 2020, 2021a).

The 5 nitazenes featured in this case report series have also been identified in Europe in 2020 (etodesnitazene) and 2023 (all others). The 5 reported postmortem cases from the U.K. where *N*-desethyl isotonitazene was identified, were collected between July and October 2023. This is in line with a recent report on the identification of *N*-desethyl isotonitazene in the urine of 19 polydrug users who presented to a hospital in Birmingham, U.K., between July and October 2023. In those cases, the patients were each unaware that they had taken a nitazene (Pucci et al. 2024). Furthermore, the detection of *N*-pyrrolidino protonitazene in 6 cases that originated from the U.K. aligns with the recent reports of its involvement in various postmortem and overdose cases in Ireland (Killeen et al. 2024; Killoran et al. 2024; HSE 2024; Burnhill 2024). In addition, to the best of the authors’ knowledge, this study is the first to identify *N*-pyrrolidino metonitazene in forensic casework from the U.K. Interestingly, while both 5-methyl etodesnitazene ('etomethazene') and ethyleneoxynitazene (Vandeputte et al., manuscript submitted) were notified to the EMCDDA EWS in Q1 2023, followed by 6-methyl etodesnitazene, fluetonitazene, and *N*,*N*-dimethyl etonitazene in Q2 2024 (EMCDDA), only 5-methyl etodesnitazene has been reported by CFSRE’s NPS Discovery program in Q1 2024 (Krotulski et al. 2024). Additionally, methylenedioxynitazene has been detected by CFSRE’s NPS Discovery program in Q2 2024, while it has not been reported in Europe at the time of writing (Q2 2024). Various factors such as differences in reporting systems, supply chain and consumer preferences may account for this observed (and likely temporary) disparity between Europe and North America.

Polydrug use has become prevalent in forensic toxicology casework, and single-drug intoxications are increasingly rare. The cases discussed in this study exemplify this trend. The evaluated nitazenes were commonly detected alongside other central nervous system (CNS) depressants, such as opioids (mainly fentanyl) and benzodiazepines (primarily NPS) (Laing et al. 2021; Krotulski et al. 2022; Vandeputte et al. 2022b). Xylazine was identified in approximately one third of cases, typically alongside fentanyl, indicating that it is frequently encountered in combination with fentanyl as an adulterant, rather than being a common nitazene adulterant. In addition, CNS stimulants such as methamphetamine, amphetamine, and cocaine were also commonly detected (Friedman and Shover 2023).

The observed blood concentrations for etodesnitazene, *N*-desethyl etonitazene, *N*-desethyl isotonitazene, *N*-pyrrolidino metonitazene, and *N*-pyrrolidino protonitazene were generally in the low or sub-ng/mL range and are similar to those reported for other nitazenes (Krotulski et al. 2020, 2021b; Vandeputte et al. 2022b, e). The low concentrations are in line with what could be anticipated from the in vitro pharmacological characterization and underscore the importance of highly sensitive analytical methods during laboratory analysis. Notably, some cases with higher concentrations were identified as well. For example, concentrations for *N*-pyrrolidino protonitazene in 3 cases (**cases 50**, **51**, and **64**) were considerably higher (range: 52–55 ng/mL) than the other cases where *N*-pyrrolidino protonitazene was quantified (range: 0.3–15 ng/mL). A similar observation was made for *N*-pyrrolidino metonitazene when comparing **case 67** (26 ng/mL) with the other *N*-pyrrolidino metonitazene cases (range: 0.2–0.9 ng/mL). Furthermore, the concentration range for etodesnitazene (0.1–120 ng/mL) is slightly broader than the ranges observed for the other 3 analogues (0.2–55 ng/mL), with higher concentrations observed in various etodesnitazene cases. This may be attributed to the at least 4 times lower potency of etodesnitazene compared to the other analogues, as shown in our in vitro MOR activation assays. However, conclusions should be drawn with caution due to variations in sample size and blood sampling site (Sastre et al. 2017). In addition, the interpretation of in vivo concentrations is complicated by various factors, including interindividual differences in opioid tolerance (Gerostamoulos et al. 2016; Vandeputte et al. 2022e) and whether the users were knowingly using nitazenes or not. The latter is particularly relevant, as nitazenes have been identified in illicit supplies of tablets being sold to contain diazepam (Martin et al. 2023), posing an evident danger of fatal accidental overdose to unsuspecting users.

As the number of non-fentanyl NSOs emerging on the drug market continues to increase, further diversification of the nitazene class is probable (Vandeputte et al., manuscript submitted). The herein newly characterized nitazenes (i.e., additional pyrrolidino, piperidine, and *N*-desethyl analogues) as well as other nitazenes that have not yet been identified on the recreational drug market (e.g., *N*-pyrrolidino isotonitazene and *N*-piperidinyl isotonitazene), can be considered the next logical candidates, or ‘prophetic’ nitazenes as some refer to it. The comparative pharmacological evaluations carried out in this study, combined with the identification and quantification of several of these newer nitazenes in forensic casework, may contribute to raising awareness, as well as strengthen preparedness and harm reduction efforts. This is necessary, as nitazenes are presenting a growing threat to public health in Europe and elsewhere (Holland et al. 2024; Giraudon et al. 2024). Further monitoring and evaluation of emerging NSO trends will remain essential to keep up with a dynamic and constantly evolving NPS market.

## Conclusion

In this study, we performed comparative in vitro pharmacological characterization of a diverse panel of known and previously uncharacterized nitazenes. This allowed us to further build on known SAR trends within the nitazene class, focusing on the structural modifications observed in nitazenes that have recently emerged on the NPS markets in the U.S. and around the world. Furthermore, we present a forensic case series including detections of etodesnitazene, *N*-desethyl isotonitazene, *N*-desethyl etonitazene, *N*-pyrrolidino metonitazene, and *N*-pyrrolidino protonitazene from medicolegal death investigations in North America and the U.K. Consistent with previous findings for other nitazenes, our data show that the in vivo blood concentrations of these drugs were generally in the low-to-sub-ng/mL range, underscoring the need for sensitive detection techniques. Given the high potencies and efficacies of many nitazenes, continuous monitoring and evaluation of this dangerous class of NSOs is warranted.

### Supplementary Information

Below is the link to the electronic supplementary material.

Supplementary file1 (DOCX 845 KB)

## Data Availability

Data can be made available upon request.
