# Supplementary material for: In vitro structure–activity relationships and forensic case series of emerging 2-benzylbenzimidazole ‘nitazene’ opioids
Source: Arch Toxicol. 2024 Jun 14;98(9):2999–3018. doi: 10.1007/s00204-024-03774-7 (PMC11324687; doi:10.1007/s00204-024-03774-7)
Supplement: Supplementary file 1 — Supplementary file1 (DOCX 845 KB) [file 204_2024_3774_MOESM1_ESM.docx]

**In vitro structure-activity relationships and forensic case series of emerging 2-benzylbenzimidazole ‘nitazene’ opioids**

Liam M. De Vrieze^1,#^, Sara E. Walton^2,#^, Eline Pottie^1^, Donna Papsun^3^, Barry K. Logan^2,3^, Alex J. Krotulski^2^, Christophe P. Stove^1,*^, Marthe M. Vandeputte^1,*^

^1^Laboratory of Toxicology, Department of Bioanalysis, Faculty of Pharmaceutical Sciences, Ghent University, Ghent, Belgium.

^2^Center for Forensic Science Research and Education, Fredric Rieders Family Foundation, Willow Grove, PA 19090, U.S.

^3^NMS Labs, Horsham, PA 19044, U.S.

^#,*^Contributed equally

*Corresponding authors: [christophe.stove@ugent.be](mailto:christophe.stove@ugent.be); [marthe.vandeputte@ugent.be](mailto:marthe.vandeputte@ugent.be)

**SUPPLEMENTARY INFORMATION**

**Table of Contents:**

[***S1: Cloning of the expression vector containing the human MOR coding sequence*** 2](#_Toc161763128)

[***S2: Calculation of efficacies relative to DAMGO in the MOR-βarr2 assay*** 3](#_Toc161763129)

[***S3: GloSensor® cAMP control experiments*** 5](#_Toc161763130)

[***S4: Efficacy values relative to DAMGO*** 6](#_Toc161763131)

[***S5: Graphic overview of the relative potency ratios of individual compounds to fentanyl*** 7](#_Toc161763132)

[***S6: Forensic toxicology cases*** 8](#_Toc161763133)

[***References*** 23](#_Toc161763134)

# ***S1: Cloning of the expression vector containing the human MOR coding sequence***

The pNBe2 plasmid containing the MOR-SmBiT construct (kindly gifted by Dr. Andy Chevigné) was adjusted through deletion of the sequences encoding SmBiT (part of the NanoBiT® system) and the linker (GSSGGGGSGGGGSSG) using the Phusion™ site-directed mutagenesis kit (Thermo Fisher Scientific, Pittsburgh, PA, U.S.), according to the manufacturer’s instructions. In short, a Mastercycler Nexus Thermal Cycler (Eppendorf, Hamburg, Germany) was used for a 3-step polymerase chain reaction (PCR) cycling protocol: initial denaturation (98 °C, 30 s), 25 cycles of denaturation (98 °C, 10 s), annealing (70.5 °C, 30 s), elongation (72 °C, 150 s), followed by a final extension (72 °C, 5 min). This PCR reaction was performed on 100 pg of plasmid DNA using the provided Phusion High Fidelity Mastermix with 0.5 μM of the forward (TAATCTAGAGTCGACCTGCAGGCATGC, *T*_m_ = 72.4 °C) and reverse (GGGCAACGGAGCAGTTTCTGCTTCCAG, *T*_m_ = 69.5 °C) primers, synthesized by Eurofins Genomics (Ebersberg, Germany). Following gel electrophoresis of the product, the PCR-amplified construct was extracted using the E.Z.N.A.® MicroElute Gel Extraction kit (VWR International, Radnor, PA, U.S.). Next, the amplification product was ligated with T4 DNA Ligase in rapid ligation buffer and heat-shock transformed into chemically competent *Escherichia coli* bacteria. After plasmid isolation with the E.Z.N.A.® Plasmid DNA Mini kit (VWR International), the construct was sequence-verified via Sanger sequencing performed by Eurofins Genomics.

# ***S2: Calculation of efficacies relative to DAMGO in the MOR-βarr2 assay***

To allow a comparative evaluation of the efficacies to DAMGO in the MOR-βarr2 assay, DAMGO (**D**) was run in a separate set of experiments together with hydromorphone (**A**) and fentanyl (**B**). The obtained potency (EC_50_ and pEC_50_) and efficacy values (E_max_) are shown in **Table S2-1**. Note that for DAMGO trifluoroacetate salt (**D**) the unknown stoichiometry of the commercially available product (DAMGO.*X*CF_3_COOH – the supplier, Cayman Chemical, was contacted but could not conclusively confirm what ‘X’ would be) precluded the calculation of EC_50_ values – hence only the efficacy (E_max_, obtained via a three-parameter fit) was calculated. The potency and efficacy data for hydromorphone (**A**) and fentanyl (**B**), determined in the initial set of experiments (i.e., corresponding to the data shown in **Table 1**), are repeated in **Table S2-2**. While it is known that, owing to some experimental and biological variation inherent to the use of cell-based assays, results between independent sets of experiments (using different freezings of the same stable cell line) may somewhat differ (Vandeputte et al. 2022b), the newly obtained data for hydromorphone and fentanyl were very well in line with those of the initial set of experiments (**Table S2-1** and **Table S2-2/Table 1**), and also in line with what we published before (e.g.,(Vasudevan et al. 2020; Vandeputte et al. 2020, 2021, 2022a)). Moreover, in line with published literature (Cannaert et al. 2018; Tsai et al. 2024), the efficacy of DAMGO exceeded that of both hydromorphone and fentanyl. From these experiments, it was possible to derive a *calculated* efficacy for DAMGO for the initial set of experiments, using a conversion factor that was calculated as following: E_max, fentanyl_ **_Table S2-2_**/E_max, fentanyl_ **_Table S2-1_** (= 170%/192%). This yielded a *calculated* E_max_ (95% CI) of 207% (203%-212%) for DAMGO, which is reported in **Table 1** and depicted as a dotted line in **Figure 3**.

|  | **EC_50_ (nM)** | **pEC_50_** | **E_max_ (% HM)** |
| --- | --- | --- | --- |
| **A. Hydromorphone** | 47.9 (38.6-59.2) | 7.32 (7.23-7.41) | 100 (96.5-104) |
| **B. Fentanyl** | 30.7 (23.8-31.8) | 7.51 (7.40-7.62) | 192 (185-200) |
| **D. DAMGO** | / | / | 234 (229-239) |

**Table S2-1.** Summary of potency (EC_50_ and pEC_50_) and efficacy (E_max_, relative to hydromorphone) measures for hydromorphone, fentanyl, and DAMGO, determined in a separate set of experiments in the MOR-βarr2 recruitment assay (*n* = 3). 95% confidence intervals (CI) are shown between parentheses.

**Table S2-2.** Overview of potency (EC_50_ and pEC_50_) and efficacy (E_max_, relative to hydromorphone) measures for hydromorphone and fentanyl as obtained in the initial set of experiments in the MOR-βarr2 recruitment assay (*n* ≥ 3) and as reported in **Table 1**. 95% confidence intervals (CI) are shown between parentheses.

|  | **EC_50_ (nM)** | **pEC_50_** | **E_max_ (% HM)** |
| --- | --- | --- | --- |
| **A. Hydromorphone** | 49.7 (39.8-61.7) | 7.30 (7.21-7.40) | 100 (96.3-104) |
| **B. Fentanyl** | 25.7 (19.2-34.6) | 7.59 (7.46-7.72) | 170 (163-178) |

# ***S3: GloSensor® cAMP control experiments***

To evaluate the potential contribution of aspecific (non-MOR-mediated) signals to the observed effects in the GloSensor® cAMP assay, a separate set of control experiments was conducted (**Fig. S3**), in which HEK 293T cells were transfected with pcDNA3.1 control plasmid DNA instead of MOR plasmid DNA, cfr. Materials and Methods. Statistical analysis of the data (mean and standard deviation of the computed AUC values) was conducted using GraphPad Prism 9 software (San Diego, CA, U.S.). Statistical significance (P values < 0.05) was determined using the Brown-Forsythe (F(58, 91) = [5.375], P < 0.0001) and Welch’s ANOVA (F(58, 43) = [2.870], P = 0.0002) tests followed by Dunnett’s T3 multiple comparisons test. *N*-Pyrrolidino etodesnitazene (**11**) was the only opioid that consistently showed non-MOR-mediated signals distinct from the control condition (i.e., cells treated with only 500 nM forskolin) in three independent experiments for the highest concentration (10 µM) (P = 0.0132). Although also at 1 µM an increase was observed, this was not significantly different from the control condition (P = 0.0536). Intriguingly, neither the highly related *N*-pyrrolidino metodesnitazene (**5**), nor the other evaluated compounds, exhibited this non-MOR-mediated activity. Evaluation of the basis of the non-specific effect observed at the highest concentration of *N*-pyrrolidino etodesnitazene was beyond the scope of this study. However, this observation did lead to the exclusion of the 10 µM data points obtained for *N*-pyrrolidino etodesnitazene from the concentration-response plots.

**Fig. S3.** Time−luminescence profiles of *N*-pyrrolidino metodesnitazene (**5**) (left panel) and *N*-pyrrolidino etodesnitazene (**11**) (right panel) in the GloSensor® cAMP control experiments. Data are from one representative experiment, in which each condition was measured in duplicate, from a total of three independent experiments (*n* = 3).

# ***S4: Efficacy values relative to DAMGO***

**Table S4.** Overview of the efficacy (E_max_, relative to DAMGO) values for all nitazenes (**1-25**) and comparator drugs (**A-D**), determined in the MOR-βarr2 recruitment assay and GloSensor® cAMP assay (*n* ≥ 3). 95% confidence intervals are shown between parentheses.

|  | **MOR-βarr2** | **GloSensor® cAMP** |
| --- | --- | --- |
|  | **E_max_ (% DAMGO)^a^** | **E_max_ (% DAMGO)^a^** |
| **1. Metonitazene** | 94.6 (88.3-101) | 95.2 (90.4-101) |
| **2. *N*-Pyrrolidino metonitazene** | 84.0 (79.2-89.3) | 98.1 (91.8-104) |
| **3. *N*-Piperidinyl metonitazene** | 73.8 (68.5-78.7) | 97.1 (90.2-104) |
| **4. Metodesnitazene** | 72.4 (66.6-78.2) | 94.4 (85.1-104) |
| **5. *N*-Pyrrolidino metodesnitazene** | 59.8 (53.1-67.1) | 99.0 (86.8-111) |
| **6. *N*-Desethyl metonitazene** | 102 (95.6-109) | 100 (94.1-107) |
| **7. Etonitazene** | 94.1 (88.8-99.9) | 101 (96.2-106) |
| **8. *N*-Pyrrolidino etonitazene** | 96.0 (91.2-101) | 102 (94.3-110) |
| **9. *N*-Piperidinyl etonitazene** | 99.9 (93.6-106) | 99.0 (94.7-103) |
| **10. Etodesnitazene** | 91.2 (84.0-98.5) | 96.2 (87.5-105) |
| **11. *N*-Pyrrolidino etodesnitazene** | 84.9 (80.6-89.8) | 92.3 (80.5-105) |
| **12. *N*-Desethyl etonitazene** | 117 (108-126) | 96.2 (90.0-103) |
| **13. Protonitazene** | 93.6 (88.3-99.4) | 102 (89.7-115) |
| **14. *N*-Pyrrolidino protonitazene** | 95.6 (93.6-97.5) | 104 (95.0-112) |
| **15. *N*-Piperidinyl protonitazene** | 96.0 (93.6-98.0) | 103 (95.2-110) |
| **16. Protodesnitazene** | 84.5 (81.1-87.8) | 104 (93.6-114) |
| **17. *N*-Desethyl protonitazene** | 108 (98.5-117) | 101 (90.1-112) |
| **18. Isotonitazene** | 103 (98.9-107) | 100 (94.1-107) |
| **19. *N*-Pyrrolidino isotonitazene** | 104 (99.9-109) | 100 (94.5-106) |
| **20. *N*-Piperidinyl isotonitazene** | 105 (100-110) | 101 (92.4-110) |
| **21. Isotodesnitazene** | 90.7 (86.4-94.6) | 101 (92.2-110) |
| **22. *N*-Desethyl isotonitazene** | 109 (105-113) | 100 (95.2-104) |
| **23. 4’-OH nitazene** | 69.5 (63.7-75.3) | 101 (92.1-110) |
| **24. *N*-Pyrrolidino 4’-OH nitazene** | 79.6 (74.3-84.9) | 101 (91.4-110) |
| **25. *N*-Piperidinyl 4’-OH nitazene** | 84.9 (80.1-90.3) | 102 (89.8-113) |
| **A. Hydromorphone** | 48.3 (46.5-50.2) | 95.2 (87.4-104) |
| **B. Fentanyl** | 82.1 (78.7-85.9) | 101 (89.1-112) |
| **C. Morphine** | 55.0 (52.6-57.4) | 95.2 (83.2-109) |
| **D. DAMGO** | 100 (98.0-102) | 100 (90.0-111) |

^a^To express efficacies of all compounds relative to DAMGO, the E_max_ values normalized to hydromorphone (**Table 1**) were transformed using the following conversion factor: E_max,HM_/E_max,DAMGO_ (= 100%/207% for the MOR-βarr2 data; = 100%/105% for the GloSensor® cAMP data).

# **
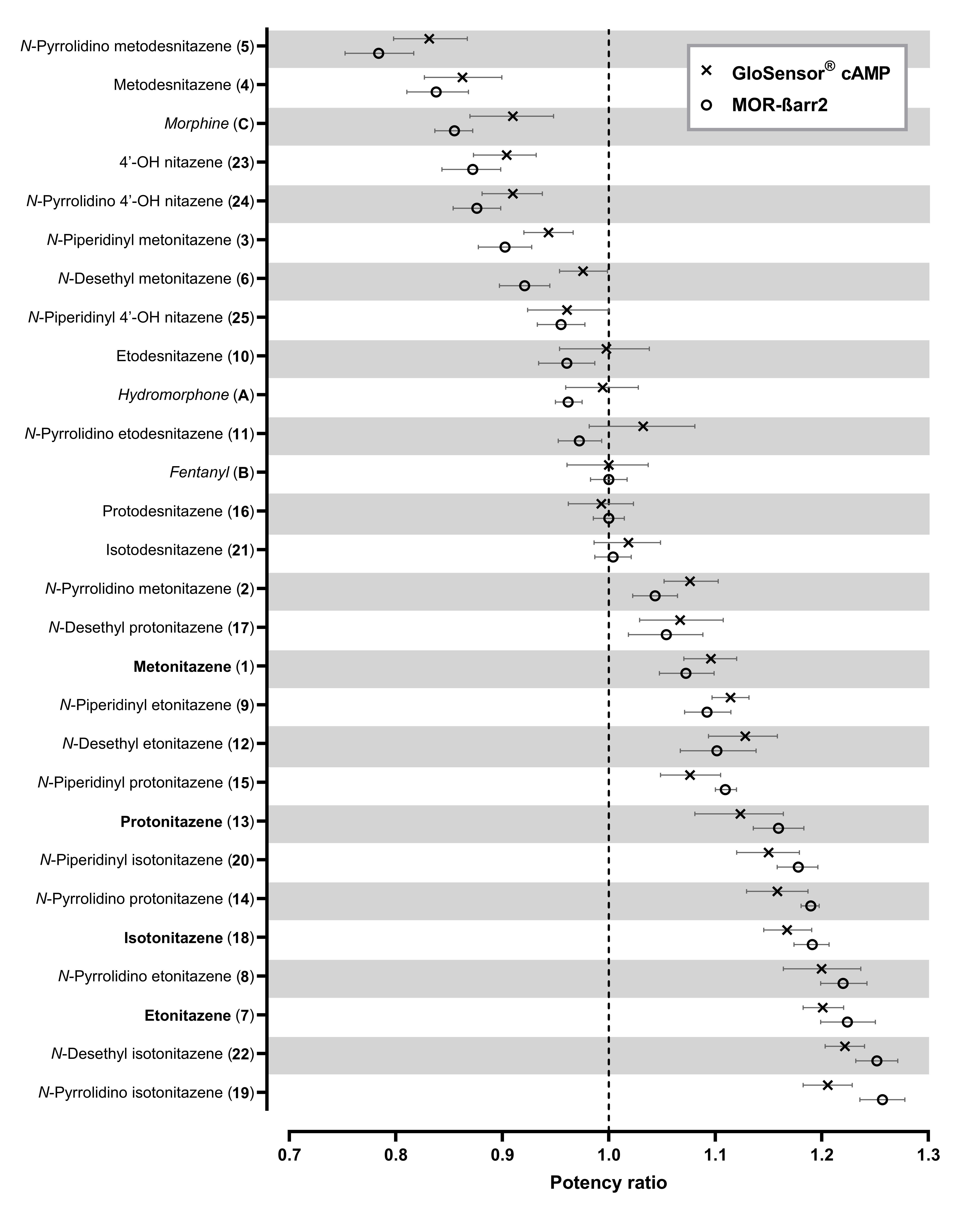
*S5: Graphic overview of the relative potency ratios of individual compounds to fentanyl***

**Fig. S5.** Visual representation of the potency data in **Table 1**, displayed as the ratio of the potency (pEC_50_) of the evaluated opioids (**1-25**, **A-C**) (with corresponding 95% confidence intervals) *versus* fentanyl (**B**). Compounds are represented by circles (MOR-βarr2 recruitment data) and crosses (GloSensor® cAMP data), with ratios >1 (<1) indicating that the compound has a higher (lower) potency than fentanyl. Reference nitazenes are highlighted in bold and comparator drugs are indicated in italics.

# ***S6: Forensic toxicology cases***

**Table S6-1.** Summary of forensic toxicology cases involving etodesnitazene.

|  | **Case description** | **Date collected** | **Date received** | **City, State** | **Matrix** | **Follow-up LC-QQQ-MS quantification of nitazenes [ng/mL]** | | **Additional toxicology results ([ng/mL] if available and unless otherwise stated)** |
| --- | --- | --- | --- | --- | --- | --- | --- | --- |
|  |  |  |  |  |  | **Etodes-nitazene** | **Other nitazenes** |  |
| **1** | 32 y/o M | 07/05/2020 | 11/07/2020 | Vancouver, BC | Femoral Blood | **Positive** | - | 4-ANPP, Acetyl Fentanyl **(0.61)**, Benzoylecgonine **(1800)**, Caffeine, Cocaine **(520)**, Fentanyl **(30)**, Flualprazolam **(11)**, Morphine **(5.2)**, Norfentanyl **(3.6)** |
| **2** | 29 y/o M, suspected OD | 18/01/2021 | 20/01/2021 | Peoria, IL | Peripheral Blood | **Positive** | - | 7-Amino Clonazepam **(18)**, Amphetamine **(10)**, Caffeine, Citalopram / Escitalopram **(440)**, Clonazepam **(2.3)**, Clonazolam **(13)**, Cotinine, Delta-9 THC **(1.2)**, Ethanol **(44 mg/dL)**, Naloxone, Nicotine |
| **3** | Suspected OD | 01/03/2021 | 03/03/2021 | Kingston, NY | Femoral Blood | **Positive** | Metonitazene* | Buproprion **(54)**, Caffeine, Cotinine, Flualprazolam **(5.7)** |
| **4** | 29 y/o M | 04/04/2021 | 13/04/2021 | Amarillo, TX | Femoral Blood | **Positive** | Isotonitazene **(Positive)** | 8-Aminoclonazolam **(47)**, Amphetamine **(100)**, Cotinine, Ethanol **(45 mg/dL)**, Mirtazapine **(71)**, Mitragynine **(750)** |
| **5** | 24 y/o M | 08/04/2021 | 13/04/2021 | Ankeny, IA | Femoral Blood | **Positive** | Isotonitazene **(Positive)**, Metonitazene **(Positive)** | 7-Amino Clonazepam **(5.6)**, Alpha-Hydroxyetizolam **(9.8)**, Alprazolam **(10)**, Amphetamine **(3400)**, Delta-9 Carboxy THC **(7.2)**, Delta-9 THC **(2.8)**, Ephedrine **(28)**, Etizolam **(13)**, Methamphetamine **(47000)**, Phenylpropanolamine **(160)** |
| **6** | 36 y/o F | 26/04/2021 | 28/04/2021 | Morgantown, WV | Urine | **Positive** | - | None Detected |
| **7** | 22 y/o M | 10/05/2021 | 12/05/2021 | Ankeny, IA | Femoral Blood | **30** | - | 3-Fluorophenmetrazine, Alpha-Hydroxyetizolam **(14)**, Amphetamine **(370)**, Benzoylecgonine **(140)**, Caffeine, Cotinine, Desmethylsertraline **(1200)**, Ethanol **(12 mg/dL)**, Etizolam **(12)**, Gabapentin **(6.7)**, Mitragynine **(310)**, Olanzapine **(38)**, Sertraline **(1000)** |
| **8** | 28 y/o M with history of illicit and prescription drug use, suspected OD | 11/06/2021 | 15/06/2021 | Ramsey, MN | Femoral Blood | **Positive** | - | 11-Hydroxy Delta-9 THC **(8.3)**, 8-Aminoclonazolam **(47)**, Alpha-hydroxyetizolam **(10)**, Bromazolam **(2.2)**, Caffeine, Etizolam **(17)** |
| **9** | 41 y/o M | 11/06/2021 | 12/06/2021 | Baton Rouge, LA | Cardiac Blood | **1.8** | - | Benzoylecgonine **(3100)**, Caffeine, Cocaethylene **(260)**, Cocaine **(1500)**, Cotinine, Delta-9 Carboxy THC **(6.8)**, Delta-9 THC **(9.0)**, Doxepin **(110)**, Ethanol **(47 mg/dL)**, Naloxone |
| **10** | 40 y/o M with history of drug use, manner of death: homicide | 07/07/2021 | 09/07/2021 | Charleston, WV | Femoral Blood | **69** | - | Benzoylecgonine **(300)**, Caffeine, Cocaethylene **(64)**, Cocaine **(150)**, Ethanol **(195 mg/dL)**, Nicotine |
| **11-1** | Suspected OD | 06/09/2021 | 08/09/2021 | Albany, NY | Cardiac Blood | **4** | - | 4-ANPP, Alpha-Hydroxyalprazolam **(7.7)**, Alprazolam **(72)**, Benzoylecgonine **(1700)**, Cocaine **(34)**, Codeine, Cotinine, Fentanyl **(11)**, Levamisole, Methamphetamine, Morphine **(13)**, Norcocaine, Norfentanyl **(4.4)**, Quinine, Xylazine |
| **11-2** |  |  |  |  | Urine | **27** | - | 4-ANPP, 6-Monoacetylmorphine **(9.7)**, 7-Amino Clonazepam **(12)**, Alpha-Hydroxyalprazolam **(670)**, Alprazolam **(180)**, Benzoylecgonine **(41000)**, Cocaethylene, Cocaine **(4100)**, Codeine, Cotinine, Fentanyl **(160)**, Levamisole, Lidocaine, Methamphetamine, Morphine **(130)**, Norcocaine, Norfentanyl **(190)**, Nicotine, *para*-Fluorofentanyl, Quinine, Xylazine |
| **12** | 37 y/o M | 03/09/2021 | 13/09/2021 | Concord, NH | Femoral Blood | **120** | - | Amlodipine, Amphetamine **(76)**, Caffeine, Citalopram / Escitalopram **(860)**, Cotinine, Desalkylflurazepam **(770)**, Diphenhydramine **(190)**, Doxylamine, Mitragynine **(190)** |
| **13** | M, unwitnessed death; evidence of drug paraphernalia and ethanol swabs at scene | 20/12/2021 | 30/12/2021 | Vancouver, BC | Subclavian Blood | **2.8** | - | 4-ANPP, Amphetamine **(260)**, Benzoylecgonine **(360)**, Caffeine, Cocaethylene, Cocaine, Cotinine, Diazepam, Ephedrine **(13)**, Ethanol **(66 mg/dL)**, Fentanyl **(9.1)**, Levamisole, Methamphetamine **(4200)**, Naloxone, Norcocaine, Nordiazepam, Norfentanyl **(1.3)**, Phenacetin, Phenylpropanolamine **(42)**, Xylazine |
| **14-1** | 41 y/o M with history of anxiety, depression, insomnia. Known medications: omeprazole, clonidine, gabapentin, amphetamine salts, famotidine, trazodone, montelukast, nifedipine, mupirocin, fluticasone inhaler | 03/01/2022 | 06/01/2022 | Reno, NV | Peripheral Blood | **55** | - | Caffeine, Flualprazolam **(10)**, Flubromazolam **(5.4)**, Lidocaine, Mitragynine **(1400)**, Monoethylglycinexylidide |
| **14-2** |  |  |  |  | Central Blood | **230** | - | Deschloroketamine, Flualprazolam, Flubromazolam, Fluoroethamphetamine, Lidocaine, Monoethylglycinexylidide, Pheniramine, Tramadol, Trazodone, Mitragynine |
| **14-3** |  |  |  |  | Urine | **210** | - | 7'-Hydroxymitragynine, 8-Aminoclonazolam, Alpha-PHP/Alpha-PiHP, Clonidine, Deschloroketamine, Eutylone, Flualprazolam, Flubromazolam, Fluoroethamphetamine, Lidocaine, mCPP  , Monoethylglycinexylidide, MXPr, *N*-Ethyl Pentylone, *O*-Desmethyltramadol, Pheniramine, Tramadol, Trazodone |
| **15** | 46 y/o M, unwitnessed death, needle marks on arms; found collapsed beside a parked car | 26/10/2021 | 30/10/2021 | Vancouver, BC | Subclavian Blood | **11** | *N*-Pyrrolidino Etonitazene **(2.8)**, Metonitazene **(1.8)** | Benzoylecgonine **(240)**, Caffeine, Cocaethylene **(68)**, Cocaine **(24)**, Cotinine, Diazepam **(160)**, Ethanol **(288 mg/dL)**, Fentanyl **(20)**, Nordiazepam **(310),** Norfentanyl **(0.45)**, Phenacetin |
| **16** | 22 y/o F, unwitnessed death, found unresponsive on bed; drug paraphernalia at scene | 05/11/2021 | 19/11/2021 | Vancouver, BC | Aortic Blood | **0.53** | - | 4-ANPP, Caffeine, Carfentanil **(0.28)**, Cotinine, Delta-9 Carboxy THC **(28)**, Delta-9 THC **(2.6)**, Fentanyl **(17)**, Naloxone, Norfentanyl **(2.0)**, Phenacetin |
| **17** | 63 y/o M with history of alcohol and illicit drug use, unwitnessed death; evidence of drug paraphernalia at scene | 21/12/2021 | 30/12/2021 | Vancouver, BC | Femoral Blood | **1.9** | Metonitazene **(5.1)** | 4-ANPP, Caffeine, Delta-9 Carboxy THC **(7.0)**, Fentanyl **(61)**, Flualprazolam **(11)**, Methadone, Nicotine, Norfentanyl **(5.6)**, Xylazine **(13)** |
| **18-1** | 26 y/o F, manner of death: accident | 23/10/2021 | 05/11/2021 | Santa Cruz, CA | Peripheral Blood | **72** | - | 1-Hydroxymidazolam **(>5000)**, Amphetamine **(300)**, Benzoylecgonine **(14000)**, Cocaethylene **(610)**, Cocaine **(780)**, Fentanyl **(63)**, Methamphetamine **(5400)**, Naloxone, Nicotine, Norfentanyl **(17)** |
| **18-2** |  |  |  |  | Urine | **68** | - | 1-Hydroxymidazolam, Cocaethylene, Cocaine, Fentanyl, Methamphetamine, Midazolam, Norcocaine, Naloxone |
| **19** | 52 y/o M with history of HIV positivity and illicit drug use, sudden death; evidence of drug paraphernalia and plant material at scene | 31/03/2022 | 07/04/2022 | Vancouver, BC | Femoral Blood | **5.2** | - | 4-ANPP, Amphetamine **(15)**, Caffeine, Diphenhydramine, Fentanyl **(38)**, Flubromazepam **(42)**, Methadone, Methamphetamine **(130)**, Norfentanyl **(4.4)**, *N*-Propionyl Norfentanyl, Phenacetin, Xylazine **(19)** |
| **20** | 32 y/o M with history of illicit drug and alcohol use, sudden death | 25/07/2022 | 09/08/2022 | Vancouver, BC | Cardiac Blood | **2.3** | Metonitazene **(1.1)** | Benzoylecgonine **(1000)**, Caffeine, Cocaethylene **(50)**, Cocaine **(54)**, Diazepam **(67)**, Ethanol **(246 mg/dL)**, Fentanyl **(7.7)**, Methamphetamine, Naloxone, Norcocaine, Nordiazepam **(140)**, Phenacetin, Xylazine |
| **21** | 48 y/o M, sudden death; evidence of drug paraphernalia at scene | 27/07/2022 | 09/08/2022 | Vancouver, BC | Cardiac Blood | **1.7** | Metonitazene **(1.0)** | 11-Hydroxy Delta-9 THC **(3.6)**, Caffeine, Delta-9 Carboxy THC **(73)**, Delta-9 THC **(11)**, Ethanol **(308 mg/dL)**, Fentanyl **(5.2)**, Methamphetamine **(7.0)** |
| **22-1** | 39 y/o M, suspected OD | 18/07/2022 | 05/08/2022 | Mount Holly, NJ | Cardiac Blood | **0.1** | - | 7-Aminoclonazepam, 7-Hydroxymitragynine, 8-Aminoclonazolam, Bromazolam, Caffeine, Cyclobenzaprine, Diphenhydramine, Etizolam, Fentanyl **(8.8)**, Hydroxyzine **(98)**, Lamotrigine **(0.54)**, Norfentanyl **(2.1)**, Mitragynine **(340)**, Yohimbine |
| **22-2** |  |  |  |  | Urine | **1.1** | - | 4-ANPP, 7-Aminoclonazepam, 7-Hydroxymitragynine, 8-Aminoclonazolam, Bromazolam, Cyclobenzaprine, Diphenhydramine, Etizolam, Fentanyl, Hydroxyzine, Lamotrigine, Lidocaine, Mitragynine, Norfentanyl |
| **23** | 19 y/o, white M | 14/12/2021 | 21/12/2021 | Harvey, LA | Subclavian Blood | **Positive** | - | 4-ANPP, Buproprion **(300)**, Caffeine, Citalopram / Escitalopram **(1200)**, Cotinine, Dextrorphan / Levorphanol **(2.4)**, Dextro / Levo Methorphan **(160),** Fentanyl **(53)**, Flubromazolam **(8.8)**, Hydroxybuproprion **(2000)**, Mitragynine **(100)**, Nicotine, Nordiazepam **(520)**, Norfentanyl **(2.9)**, Promethazine **(31)** |
| **24** | 29 y/o M with history of depression and suicide attempt, unwitnessed death; evidence of drug paraphernalia at scene | 24/01/2022 | 07/02/2022 | Vancouver, BC | Cardiac Blood | **Positive** | - | 4-ANPP, Acetyl Fentanyl **(0.50)**, Amphetamine **(61)**, Caffeine, Cotinine, Fentanyl **(12)**, Flualprazolam **(7.1)**, Methamphetamine **(1200)**, Methocarbamol **(2.0)**, Nicotine, Norfentanyl **(2.4)** |
| **25** | M | Not provided | 07/12/2022 | Farmington, CT | Peripheral Blood | **Positive** | - | Alpha-Hydroxyetizolam **(6.6)**, Cotinine, Delta-9 THC **(1.4)**, Mitragynine **(390)**, Naloxone |
| **26** | 56 y/o F with suspected fentanyl use | 19/05/2023 | 16/06/2023 | Clackamas, OR | Cardiac Blood | **Positive** | - | Bromazolam **(220)**, Caffeine, Flubromazepam **(290)** |

**Key:** M – Male, F – Female, IL – Illinois, NY – New York, TX – Texas, IA – Iowa, WV – West Virginia, MN – Minnesota, LA – Louisiana, NH – New Hampshire, NV – Nevada, CA – California, NJ – New Jersey, CT – Connecticut, BC – British Columbia, OR – Oregon, OD – overdose, HIV – human immunodeficiency virus, THC – tetrahydrocannabinol, 4-ANPP – 4-anilino-*N*-phenethylpiperidine, Alpha-PHP/Alpha-PiHP – alpha-pyrrolidinohexanophenone/[alpha-pyrrolidinoisohexanophenone](https://www.ncbi.nlm.nih.gov/pcsubstance/?term=%22alpha-Pyrrolidinoisohexaphenone%22%5bCompleteSynonym%5d%20AND%2059809191%5bStandardizedCID%5d), MxPr – methoxpropamine, mCPP – meta-chlorophenylpiperazine

Note: All quantitative results pertaining to the additional toxicology findings were obtained using routine methods at NMS Labs. Findings for opioids of the morphinan class are reported as aglycon concentrations. In the column ‘Other nitazenes’, results labeled with an asterisk (*) are from initial toxicology testing only. Variations in the inclusion of case descriptors such as age, sex, ethnicity, and circumstances are reflective of the available information for each case provided by medical examiners and coroners.

**Table S6-2.** Summary of forensic toxicology cases involving *N*-pyrrolidino metonitazene and *N*-pyrrolidino protonitazene.

|  | **Case description** | **Date collected** | **Date received** | **City, State** | **Matrix** | **Follow-up LC-QQQ-MS quantification of nitazenes [ng/mL]** | | | **Additional toxicology results ([ng/mL] if available and unless otherwise stated)** |
| --- | --- | --- | --- | --- | --- | --- | --- | --- | --- |
|  |  |  |  |  |  | ***N*-Pyrrolidino protonitazene** | ***N*-Pyrrolidino metonitazene** | **Other nitazenes** |  |
| **27** | 32 y/o F | 17/12/2022 | 20/12/2022 | Chicago, IL | Femoral Blood | **1.3** | - | Isotonitazene **(Positive)**, Protonitazene **(1.6)**, Metonitazene **(0.9)**, *N*-Desethyl Isotonitazene **(0.1)**, *N*-Desethyl Protonitazene **(0.8)** | 4-ANPP **(1.4)**, Alprazolam, Diphenhydramine, Fentanyl **(4.5)**, Methadone, Norbuprenorphine **(1.3)**, Norfentanyl **(2.3)**, Noscapine, *O*-Desmethyltramadol, Quetiapine, Quinine, Tramadol **(42)**, Xylazine |
| **28** | 51 y/o M, manner of death: accident | 17/12/2022 | 20/12/2022 | Ramsey, MN | Femoral Blood | **0.9** | - | Isotonitazene **(Positive)**, Protonitazene (**Positive**), Metonitazene **(2.6)**, *N*-Desethyl Isotonitazene **(0.2)**, *N*-Desethyl Protonitazene **(0.2)** | 4-ANPP **(4.8)**, 8-Aminoclonazolam **(18)**, Acetyl Fentanyl **(25)**, Amlodipine, beta-Hydroxy Fentanyl, Caffeine, Codeine, Cotinine, Diphenhydramine, EDDP **(430)**, EMDP, Fentanyl **(100)**, Hydroxybupropion, Methadone **(2600)**, Morphine **(9.7)**, Norfentanyl **(54)**, *para*-Fluorofentanyl, Quinine, Xylazine **(9.3)** |
| **29** | 43 y/o M with history of substance abuse | 16/03/2023 | 22/03/2023 | Charleston, WV | Femoral Blood | **1.1** | - | - | Amphetamine **(59)**, Bromazolam **(36)**, Cotinine, Desalkylflurazepam **(11)**, Methamphetamine **(480)** |
| **30-1** | 29 y/o M | 07/02/2023 | 10/02/2023 | Wheaton, IL | Peripheral Blood | **0.1** | - | - | 4-ANPP **(3.9)**, Benzoylecgonine **(620)**, Chlordiazepoxide, Cotinine, Diphenhydramine **(310)**, Fentanyl **(24)**, Hydroxyzine **(100)**, Naloxone, Nordiazepam, Norfentanyl **(5.7)**, Quetiapine, Quinine, Xylazine **(28)** |
| **30-2** |  |  |  |  | Cardiac Blood | **0.4** | **0.25** | Protonitazene **(Positive)**, Metonitazene **(0.63)**, *N*-Desethyl Protonitazene **(0.12)** | 4-ANPP, Benzoylecgonine, Buprenorphine, Chlordiazepoxide, Cocaine, Diphenhydramine, Fentanyl, Hydroxyzine, Naloxone, Nordiazepam, Norfentanyl, Quetiapine, Quinine, Xylazine |
| **31** | 25 y/o F with history of morbid obesity, intravenous drug use and nasal drug use | 07/03/2023 | 21/03/2023 | Charleston, WV | Subclavian Blood | **Positive** | **Positive** | Metonitazene*, Protonitazene **(Positive)**, *N*-Desethyl Protonitazene **(Positive)** | 4-ANPP **(8.4)**, Acetyl Fentanyl, Buprenorphine **(0.76)**, Caffeine, Citalopram, Cotinine, Despropionyl *para*-Fluorofentanyl, Diphenhydramine **(150)**, Fentanyl **(71)**, Lamotrigine **(0.25 µg/mL)**, Naloxone, Norbuprenorphine **(1.3)**, Norfentanyl **(3.7)**, *para*-Fluorofentanyl **(2.2)**, Quetiapine **(290)**, Quinine, Xylazine **(48)** |
| **32** | 67 y/o M, gunshot wound to chest, found with note that says ‘C-Ya’ | 11/03/2023 | 22/03/2023 | Charleston, WV | Subclavian Blood | **1.5** | **0.47** | Protonitazene **(Positive)**, Metonitazene **(2.0)**, *N*-Desethyl Protonitazene **(0.56)** | 4-ANPP **(7.8)**, Acetaminophen **(50 µg/mL)**, Caffeine, Codeine **(700)**, Delta-9 Carboxy-THC **(17)**, Delta-9 THC **(3.0)**, Diphenhydramine, Fentanyl **(60)**, Hydrocodone **(6.5)**, mCPP **(0.15 µg/mL)**, Methamphetamine, Morphine **(6.2)**, Norcodeine, Norfentanyl **(21)**, Noroxycodone, Oxycodone **(5.3)**, Quinine, Trazodone **(1.9 µg/mL)** |
| **33** | 54 y/o M | 07/03/2023 | 08/03/2023 | Chicago, IL | IVC Blood | **1.1** | **0.63** | Isotonitazene **(0.64)**, Protonitazene **(1.1)**, Metonitazene **(6.9)**, 5-Aminoisotonitazene **(Positive)**, 4'-Hydroxy Nitazene **(Positive)**, *N*-Desethyl Protonitazene **(0.91)**, *N*-Desethyl Isotonitazene **(0.25)**, *N*-Desethyl Metonitazene* | 4-ANPP **(21)**, Acetyl Fentanyl **(0.38)**, Caffeine, Cotinine, Desalkylgidazepam, Diphenhydramine **(600)**, Fentanyl **(93)**, Lidocaine, Naloxone, Nicotine, Norfentanyl **(6.1)**, Quinine |
| **34** | 29 y/o M, traffic collision | 16/03/2023 | 17/03/2023 | Joilet, IL | Cardiac Blood | **Positive** | **Positive** | Metonitazene **(Positive)** | 4-ANPP **(110)**, Acetyl Fentanyl **(2.4)**, Acrylfentanyl **(0.053)**, Butyrylfentanyl **(0.10)**, Caffeine, Carboxyhemoglobin **(3% Saturation)**, Cotinine, Delta-9 Carboxy-THC **(16)**, Delta-9 THC **(0.86)**, Diphenhydramine **(1200)**, Fentanyl **(650)**, Nicotine, Norfentanyl **(250)**, Methadone **(140)**, Xylazine **(14)** |
| **35** | 68 y/o F, suspected OD | 06/02/2023 | 08/02/2023 | Springfield, IL | Peripheral Blood | **Positive** | **Positive** | Metonitazene* | 4-ANPP **(3.4)**, Amlodipine, Benzoylecgonine **(380)**, Fentanyl **(17)**, Norfentanyl **(3.9)** |
| **36** | 75 y/o M | 11/12/2022 | 13/12/2022 | Chicago, IL | Cardiac Blood | **0.8** | **0.5** | Isotonitazene **(2.5)**, Protonitazene **(15)**, Metonitazene **(24)**, 4'-Hydroxy Nitazene **(Positive)**, *N*-Desethyl Isotonitazene **(0.2)**, *N*-Desethyl Protonitazene **(0.5)** | 4-ANPP **(43)**, 6-Monoacetylmorphine **(76)**, 8-Aminoclonazolam, Acetyl Fentanyl **(0.32)**, beta-Hydroxy Fentanyl, Caffeine, Codeine **(7.0)**, Despropionyl *para*-Fluorofentanyl, Diphenhydramine **(2300)**, Doxylamine, EDDP, Ethanol **(27 mg/dL)**, Ethyl 4-ANPP, Fentanyl **(340)**, Lidocaine, Methadone **(66)**, Morphine **(120)**, Nifedipine, Nicotine, Norfentanyl **(26)**, Noscapine, *N*-Propionyl Norfentanyl, *O*-Desmethyltramadol, Papaverine, *para*-Fluorofentanyl **(40)**, *para*-Fluoro Phenethyl-4-ANPP, PCP **(6.8)**, Phenethyl-4-ANPP, Quetiapine **(960)**, Quinine, Tramadol, Trazodone, Xylazine **(780)** |
| **37** | 32 y/o F | 14/12/2022 | 20/12/2022 | Chicago, IL | Peripheral Blood | **Positive** | - | Metonitazene **(Positive)** | 4-ANPP, Dimethylpentylone, Fentanyl, Morphine, Norfentanyl, Quinine, Xylazine |
| **38** | 63 y/o M | 03/03/2023 | 07/03/2023 | Springfield, IL | Peripheral Blood | **Positive** | - | Metonitazene* | 4-ANPP **(2.3)**, 8-Aminoclonazolam **(6.1)**, Caffeine, Cyclobenzaprine **(45)**, Desmethylsertraline **(1000)**, Fentanyl **(21)**, Norfentanyl **(5.3)**, Sertraline **(720)** |
| **39** | 26 y/o M with history of intravenous opiate use, found unresponsive in his vehicle at a gas station; drugs and drug paraphernalia (used syringe, spoon, crystalline substance) were found in the vehicle | 08/03/2023 | 09/03/2023 | Kalamazoo, MI | Iliac Blood | **Positive** | - | - | 4-ANPP **(2.2)**, Acetyl Fentanyl **(2.7)**, Benzoylecgonine **(140)**, Cocaine **(15)**, Delta-9 Carboxy-THC **(10)**, Delta-9 THC **(2.1)**, Fentanyl **(16)**, Morphine **(9.4)**, Norfentanyl **(5.0)**, *para*-Fluorofentanyl **(0.41)** |
| **40** | 42 y/o F, undetermined death; found unresponsive at residence. Medications: suboxone, ondansetron, escitalopram, metformin | 24/04/2023 | 27/04/2023 | Waukesha, WI | Iliac Blood | **Positive** | - | Metonitazene*, Protonitazene* | 4-ANPP **(1.0)**, Bromazolam **(11)**, Buprenorphine **(0.57)**, Caffeine, Citalopram/Escitalopram **(600)**, Cotinine, Fentanyl **(5.7)**, Naloxone, Norbuprenorphine **(0.52)**, Norfentanyl **(1.0)**, Quinine |
| **41** | 46 y/o F | 01/05/2023 | 03/05/2023 | Chicago, IL | IVC Blood | **1.2** | - | Protonitazene **(Positive, <0.5)**, Metonitazene **(Positive, <0.5)** | 4-ANPP **(0.29)**, Bromazolam, Caffeine, Cocaethylene, Cocaine **(95)**, Cotinine, Despropionyl *para*-Fluorofentanyl, Diphenhydramine **(130)**, Ethanol **(144 mg/dL)**, Fentanyl, Gabapentin **(5.5 µg/mL)**, Norcocaine, *para*-Fluorofentanyl **(33)**, Quinine |
| **42** | 40 y/o M, suspected OD | 12/05/2023 | 16/05/2023 | Springfield, IL | Peripheral Blood | **0.5** | **0.2** | Metonitazene **(1.2)**, Protonitazene **(Positive, <0.5)**, *N*-Desethyl Protonitazene **(0.1)** | 8-Aminoclonazolam **(8.7)**, Diphenhydramine, Naloxone, Olanzapine **(8.8)**, Quinine |
| **43** | 44 y/o F with history of cocaine and heroin use | 25/05/2023 | 26/05/2023 | McFarland, WI | Femoral Blood | **0.8** | **0.4** | Metonitazene **(2.7)**, Protonitazene **(0.72)**, *N*-Desethyl Protonitazene **(Positive)** | 8-Aminoclonazolam **(4.9)**, Acetaminophen, Amphetamine **(18)**, Benzoylecgonine **(100)**, Caffeine, Clomipramine **(270)**, Cocaine, Cotinine, Desmethylclomipramine **(69)**, Dihydrocodeine **(5.2)**, Gabapentin **(8.3)**, Hydrocodone **(5.2)**, Naloxone, Quinine |
| **44** | 37 y/o F, undetermined death | 30/05/2023 | 07/06/2023 | Sacramento, CA | Femoral Blood | **0.3** | - | - | Acetaminophen |
| **45** | 60 y/o M, single vehicle accident | 06/06/2023 | 07/06/2023 | Cambridge, IL | Cardiac Blood | **1.6** | **0.4** | Metonitazene **(2.0)**, *N*-Desethyl Protonitazene **(0.1)** | 4-ANPP **(11)**, 8-Aminoclonazolam **(6.3)**, Bipiperidinyl 4-ANPP, Caffeine, Codeine **(10)**, Cotinine, EDDP **(94)**, Fentanyl **(49)**, Methadone **(530)**, Morphine **(150)**, Nicotine, Norfentanyl **(24)**, *N*-Propionyl Norfentanyl, Quinine |
| **46** | 54 y/o M, undetermined death | 01/06/2023 | 08/06/2023 | Augusta, ME | Femoral Blood | **0.9** | - | - | 4-ANPP **(0.89)**, Amphetamine **(6.3)**, Benzoylecgonine **(340)**, Caffeine, Fentanyl **(7.4)**, Methamphetamine **(31)**, Norfentanyl **(1.8)** |
| **47** | 36 y/o M with history of heroin use, witnessed arrest | 08/06/2023 | 13/06/2023 | McFarland, WI | Femoral Blood | **1.1** | **0.7** | Metonitazene **(4.0)**, Protonitazene **(0.59)**, *N*-Desethyl Protonitazene **(0.4)** | 4-ANPP **(8.9)**, 11-Hydroxy-Delta-9 THC **(3.0)**, Cotinine, Delta-9 Carboxy-THC **(68)**, Delta-9 THC **(13)**, Diphenhydramine **(100)**, Fentanyl **(58)**, Naloxone, Norfentanyl **(15)**, *O*-Desmethyltramadol, *para*-Fluorofentanyl **(2.2)**, Promethazine **(30)**, Quinine, Tramadol, Xylazine |
| **48** | M | Not provided | 14/06/2023 | Birmingham, UK | Femoral Blood | **1** | - | - | Benzoylecgonine **(650)**, Caffeine, Cocaethylene **(16)**, Cocaine **(100)**, Cotinine, Morphine **(13)**, Naloxone, Norcocaine, Papaverine, Phenacetin |
| **49** | M | Not provided | 14/06/2023 | Birmingham, UK | Femoral Blood | **1.5** | - | - | Amitriptyline **(740)**, Amphetamine **(130)**, Benzoylecgonine **(1200)**, Buprenorphine **(0.71)**, Caffeine, Cocaethylene, Cocaine, Codeine, Cotinine, Cyclobenzaprine, Diazepam **(20)**, Levamisole, Mirtazapine **(150)**, Morphine **(17)**, Norbuprenorphine **(1.9)**, Norcocaine, Nordiazepam **(30)**, Nortriptyline **(660)**, Noscapine, Olanzapine **(170)**, Phenacetin |
| **50** | 30 y/o M with history of drug use, found dead on bed | 13/06/2023 | 20/06/2023 | Stevens Point, WI | Subclavian Blood | **55** | - | - | Bromazolam, Caffeine, Cotinine, Naloxone |
| **51** | 50 y/o M with history of heroin misuse since 2014 and substance misuse dating back to 1992 | 14/06/2023 | 21/06/2023 | Birmingham, UK | Femoral Blood | **53** | - | - | 6-Monoacetylmorphine **(19)**, Acetaminophen, Benzoylecgonine **(530)**, Caffeine, Cocaethylene **(32)**, Cocaine **(160)**, Codeine **(43)**, Cotinine, EDDP **(68)**, Ethanol **(57 mg/dL)**, Levamisole, Methadone **(1800)**, Morphine **(360)**, Nicotine, Norcocaine, Noscapine, Papaverine, Phenacetin, Pregabalin **(2.7 µg/mL)**, Quetiapine |
| **52** | None provided | Not provided | 22/06/2023 | Maynard, MA | Iliac Blood | **8.5** | - | *N*-Pyrrolidino 4'-Hydroxy Nitazene* | 4-ANPP **(2.6)**, Amphetamine **(28)**, Caffeine, EDDP **(46)**, EMDP, Fentanyl **(20)**, Methadone **(450)**, Methamphetamine **(420)**, Norfentanyl **(4.9)**, *para*-Fluorofentanyl |
| **53** | 61 y/o F found dead at home address. Medical history: previous intravenous drug use, heavy drinker | 08/06/2023 | 23/06/2023 | Birmingham, UK | Femoral Blood | **Positive** | - | - | Acetaminophen, Benzoylecgonine **(1000)**, Caffeine, Cocaethylene, Cocaine, Codeine **(5.5)**, Diazepam, Hydromorphone **(2.0)**, Levamisole, Methadone, Morphine **(260)**, Norcocaine, Noscapine, *O*-Desmethyltramadol, Papaverine, Temazepam **(66)**, Tramadol |
| **54** | None provided | Not provided | 07/07/2023 | Maynard, MA | Subclavian Blood | **Positive** | - | - | 4-ANPP **(53)**, 7-Aminoclonazepam **(23)**, Acetyl Fentanyl **(0.30)**, Benzoylecgonine **(3500)**, Cocaine **(91)**, Cotinine, Delta-9-Carboxy THC **(15)**, Delta-9 THC **(3.2)**, Fentanyl **(200)**, Morphine **(10)**, Naloxone, Norfentanyl **(14)**, *para*-fluorofentanyl **(7.5)**, Xylazine **(19)** |
| **55** | 41 y/o F | 06/07/2023 | 10/07/2023 | Chicago, IL | IVC Blood | **Positive** | - | - | 4-ANPP **(240)**, Acetyl fentanyl **(2.2)**, Benzoylecgonine **(1100)**, Caffeine, Cocaine **(170)**, Cotinine, Diphenhydramine **(2200)**, Fentanyl **(730)**, Naloxone, Nicotine, Norfentanyl **(680)**, *para*-Fluorofentanyl **(18)**, Quinine, Xylazine **(17)** |
| **56** | F | 24/07/2023 | 25/07/2023 | Toledo, OH | Heart Blood | **2.4** | - | - | 4-ANPP **(3.1)**, Acetaminophen, Cocaine, Despropionyl *para*-Fluorofentanyl, Diphenhydramine, EDDP, Fentanyl **(41)**, Lidocaine, mCPP, Methadone, Norfentanyl **(0.75)**, Noroxycodone, *N*-Propionyl Norfentanyl, Oxycodone, *para*-Fluorofentanyl **(4.5)**, Quinine, Trazodone, Xylazine |
| **57** | 55 y/o M | 25/07/2023 | 26/07/2023 | Chicago, IL | IVC Blood | **3.3** | - | Metonitazene **(0.74)** | 4-ANPP **(4.7)**, Carbamazepine, Citalopram/Escitalopram **(100)**, Diphenhydramine **(58)**, Fentanyl **(33**), Lamotrigine **(15 µg/mL)**, Nicotine, Norfentanyl **(4.6)**, Quinine |
| **58** | 44 y/o M with history of mental health issues, suicidal ideation, and illicit drug use, sudden death; evidence of drug paraphernalia and empty naloxone kit at scene. | 26/07/2023 | 01/08/2023 | Vancouver, BC | Subclavian Blood | **1.9** | - | - | 4-ANPP **(2.9)**, Amphetamine **(52)**, Bromazolam **(44)**, Cocaine, Fentanyl **(45)**, Methamphetamine **(210)**, Norfentanyl **(3.5)**, *N*-Propionyl Norfentanyl |
| **59** | None provided | Not provided | 04/08/2023 | Maynard, MA | Femoral Blood | **Positive** | - | - | Caffeine, Cotinine, Ethanol **(95 mg/dL)**, Fentanyl **(1.7)**, Naloxone |
| **60** | 44 y/o M | 11/08/2023 | 15/08/2023 | Chicago, IL | IVC Blood | **0.6** | **0.9** | Metonitazene **(1.2)** | 4-ANPP **(0.34)**, Benzoylecgonine **(2600)**, Cocaine **(77)**, Delta-9-Carboxy THC **(7.3)**, Delta-9 THC **(1.5)**, Fentanyl, Ketamine **(650)**, Lidocaine, MDMA, Norcocaine, Norketamine **(360)**, Quinine |
| **61** | 26 y/o M; found in a rural farmland, just off a public footpath, lying down fully clothed on a towel which had been carefully placed on the ground. No signs of trauma or injury. | 15/08/2023 | 17/08/2023 | Birmingham, UK | Femoral Blood | **15** | - | *N*-Pyrrolidino 4'-Hydroxy Nitazene* | 7-Aminoclonazepam **(750)**, Caffeine, Clonazepam **(35)**, Cotinine |
| **62** | 58 y/o M, suspected OD | 24/08/2023 | 25/08/2023 | Springfield, IL | Peripheral Blood | - | **Positive** | Metonitazene* | 4-ANPP **(1.7)**, 8-Aminoclonazolam **(3.6)**, Amlodipine, Brorphine **(0.50)**, Cotinine, EDDP **(190)**, Fentanyl **(21)**, Methadone **(1000)**, Norfentanyl **(5.5)** |
| **63** | 20 y/o F, suspected OD | 02/07/2023 | 29/08/2023 | Concord, NH | Antemortem Blood | **0.8** | - | - | 4-ANPP **(0.87)**, 11-Hydroxy Delta-9 THC **(1.5)**, Delta-9 Carboxy-THC **(60)**, Delta-9 THC **(4.2)**, Dextrorphan / Levorphanol **(63)**, Dextro / Levo Methorphan **(140)**, Fentanyl **(9.0)**, Naloxone, Norfentanyl **(0.66)**, Xylazine **(17)** |
| **64** | 46 y/o F with history of excessive alcohol and drug user; suffering from impaired cognition | 31/08/2023 | 06/09/2023 | Birmingham, UK | Femoral Blood | **52** | **0.3** | - | 6-Monoacetylmorphine **(1.4)**, Acetaminophen, Benzoylecgonine **(2100)**, Caffeine, Citalopram, Cocaethylene **(65)**, Cocaine **(410)**, Codeine **(10)**, Cotinine, Delta-9 THC **(2.0)**, Diazepam **(150)**, Diphenhydramine, Ethanol **(141 mg/dL)**, Eszopiclone/Zopiclone **(21)**, Levamisole, Mirtazapine **(370)**, Morphine **(110)**, Nicotine, Norcocaine, Nordiazepam **(200)**, Noscapine, Oxazepam, Papaverine, Temazepam |
| **65** | 43 y/o M, undetermined manner of death | 14/09/2023 | 22/09/2023 | Augusta, ME | Femoral Blood | **Positive** | - | - | 9-Hydroxyrisperidone **(15)**, Amphetamine **(46)**, Caffeine, Cotinine, Gabapentin **(14 µg/mL)**, Methamphetamine **(110)**, Naloxone |
| **66** | 56 y/o M with history of cocaine and heroin use | 20/09/2023 | 03/10/2023 | McFarland, WI | Peripheral Blood | **Positive** | - | - | 4-ANPP **(1.9)**, Acetyl fentanyl **(2.4)**, Caffeine, Ethanol **(83 mg/dL)**, Fentanyl **(23)**, Naloxone, Morphine **(73)**, *para*-Fluorofentanyl **(1.9)** |
| **67** | M | 05/06/2023 | 30/06/2023 | Allentown, PA | Femoral Blood | - | **26** | - | Amphetamine, Bromazolam, Naloxone, Tianeptine |

**Key:** M – Male, F – Female, IVC – Inferior vena cava, IL – Illinois, WV – West Virginia, MN – Minnesota, NH – New Hampshire, CA – California, PA – Pennsylvania, MI – Michigan, BC – British Columbia, OH – Ohio, WI – Wisconsin, ME – Maine, MA – Massachusetts, UK – United Kingdom, OD – overdose, THC – tetrahydrocannabinol, 4-ANPP – 4-anilino-*N*-phenethylpiperidine, PCP – phencyclidine, EDDP – 2-ethylidene-1,5-dimethyl-3,3-diphenylpyrrolidine, EMDP – 2-ethyl-5-methyl-3,3-diphenylpyrroline, mCPP – meta-chlorophenylpiperazine, MDMA – 3,4-methylenedioxymethamphetamine

Note: All quantitative results pertaining to the additional toxicology findings were obtained using routine methods at NMS Labs. Findings for opioids of the morphinan class are reported as aglycon concentrations. In the column ‘Other nitazenes’, results labeled with an asterisk (*) are from initial toxicology testing only. If *N*-desethyl isotonitazene and/or *N*-desethyl protonitazene were found together with isotonitazene and protonitazene, respectively, they were considered metabolites (i.e., these findings were placed in the ‘Other nitazenes’ column and the cases were not separately placed in **Table S6-3**). In case **33**, *N*-desethyl metonitazene was found together with metonitazene and was therefore also considered as a metabolite. Case **45,** where *N*-desethyl protonitazene was found in the absence of protonitazene, was excluded from **Table S6-3** as *N*-pyrrolidino metonitazene and *N*-pyrrolidino protonitazene were detected at higher concentrations. Variations in the inclusion of case descriptors such as age, sex, ethnicity, and circumstances are reflective of the available information for each case provided by medical examiners and coroners.

**Table S6-3.** Summary of forensic toxicology cases involving *N*-desethyl etonitazene*, N*-desethyl isotonitazene, *N*-desethyl protonitazene.

|  | **Case description** | **Date collected** | **Date received** | **City, State** | **Matrix** | **Follow-up LC-QQQ-MS quantification of nitazenes [ng/mL]** | | | | **Additional toxicology results ([ng/mL] if available and unless otherwise stated)** |
| --- | --- | --- | --- | --- | --- | --- | --- | --- | --- | --- |
|  |  |  |  |  |  | ***N*-Desethyl etonitazene** | ***N*-Desethyl isotonitazene** | ***N*-Desethyl protonitazene** | **Other nitazenes** |  |
| **68** | 52 y/o M with history of drug use, cause of death: toxicity by the combined effects of cocaine, methamphetamine, bromazolam, *N*-desethyl isotonitazene, and ethanol. Manner of death: accident. | 07/01/2023 | 10/01/2023 | North Brunswick, NJ | Femoral Blood | - | **0.82** | - | - | Amphetamine **(43)**, Benzoylecgonine **(3900)**, Bromazolam, Cocaethylene **(87)**, Cocaine **(470)**, Cotinine, Delta-9 THC **(0.88)**, Diphenhydramine, Methamphetamine **(21)**, Norcocaine, Phenacetin |
| **69** | 32 y/o M, driving under the influence of drugs | 05/12/2022 | 09/12/2022 | Quakertown, PA | Blood | - | **5.1** | - | Protonitazene **(Positive)** | 4-ANPP, Benzoylecgonine **(1300)**, Bromazolam **(64)**, Caffeine, Cocaine **(43)**, Ethyl 4-ANPP, Fentanyl **(6.1)**, Flubromazepam, Levamisole, Methamphetamine, Norcocaine, Norfentanyl **(2.6)**, Phenethyl 4-ANPP, Quinine, Xylazine **(93)** |
| **70** | 36 y/o white F, probable OD, was found sleeping on a couch in a house known to police department as a ‘drug house’, some 9 hours later found unresponsive on the same couch by another resident, bag of heroin reported to be under decedent; past medical history: schizophrenia and hepatitis C, admitted to using heroin and smoking one pack of cigarettes per day. No alcohol use. | 04/03/2023 | 07/03/2023 | Knoxville, TN | Cardiac Blood | - | **Positive** | - | - | Amphetamine **(59)**, Caffeine, Methamphetamine **(480)**, Naloxone, Quinine, Xylazine |
| **71-1** | 21 y/o M with history of polysubstance use, probable mixed drug intoxication; found with dried frothy fluid on face and pill labelled ‘IP204’ | 16/02/2023 | 06/04/2023 | El Paso, TX | Blood | - | **5** | - | - | Acetaminophen, Bromazolam, Lidocaine, Noroxycodone, Oxycodone |
| **71-2** |  |  |  |  | Urine | - | **1.7** | - | - | Acetaminophen, Lidocaine, Noroxycodone, Methamphetamine, Oxycodone |
| **72** | 69 y/o F, found in living room, sitting on couch; no signs of trauma; there are wax baggies next to her and some found in the bathroom; Narcan in the residence. Medical history: drug use, HIV positive, hepatitis C, hypertension, high cholesterol. Medications: oxycodone, atorvastin | Not provided | 07/12/2022 | Farmington, CT | Femoral Blood | - | **Positive** | - | - | 2-Fluoro-2-oxo-PCE, 4-ANPP **(0.82)**, 7-Amino Clonazepam **(43)**, Bromazolam, Caffeine, Fentanyl **(7.8)**, Flubromazepam **(72)**, Norfentanyl **(3.4)**, Xylazine **(13)** |
| **73** | 57 y/o F, suspected OD; found lying on side of bed; narcotics history – heroin, on methadone program. Medical history: depression and anxiety. Medications: fomotidine, fluoxetine, gabapentin, clonazepam, levothyroxine | Not provided | 23/03/2023 | Farmington, CT | Femoral Blood | - | - | **Positive** | - | 7-Amino Clonazepam, Bromazolam, Clonazepam, Diphenhydramine, Fluoxetine, Norfluoxetine |
| **74** | 50 y/o M with history of intravenous drug use, pill abuse, possible OD; copious plastic baggies, straw, scale, large packet of white powder found on scene. Last seen alive when speaking while sitting but with grey appearance and suffering from shortness of breath, and collapsed some 2 h later. | 24/06/2023 | 27/06/2023 | Knoxville, TN | Femoral Blood | - | **2.2** | - | Metonitazene **(0.6)** | 4-ANPP **(0.97)**, Acetaminophen, Alprazolam **(16)**, Bromazolam, Caffeine, Cotinine, Fentanyl **(5.1)**, Lidocaine, Naloxone, Norfentanyl **(1.0)**, Noroxycodone, Oxycodone **(97)**, Oxymorphone **(1.0)**, Quinine, Tadalafil **(39)**, Xylazine **(41)** |
| **75** | 47 y/o white M, suspicious OD; Decedent found by homeowner sitting on front porch of residence barely responsive, admitted to using heroin - 9-1-1 was called and decedent was transported to emergency department - removed clothing and found fresh 2nd degree burns on upper thighs and back, clothing not burned. | 10/07/2023 | 12/07/2023 | Knoxville, TN | Peripheral Blood | - | **2** | - | - | 4-ANPP **(0.50)**, EDDP, Fentanyl **(2.6)**, Methadone, Naloxone, Norfentanyl **(5.1)**, Quinine, Xylazine **(100)** |
| **76** | 35 y/o M, found dead by hanging | 11/07/2023 | 24/07/2023 | Stourport, UK | Femoral Blood | - | **3.4** | - | - | 6-Monoacetylmorphine **(5.7)**, Acetaminophen, Benzoylecgonine **(8000)**, Caffeine, Carbamazepine, Cocaethylene, Cocaine **(570)**, Codeine **(34)**, Cotinine, Diazepam, EDDP, Hydromorphone **(8.6)**, Methadone, Morphine **(840)**, Norcocaine, Nordiazepam **(100)**, Noscapine, Olanzapine **(100)**, Papaverine, Phenacetin |
| **77** | 35 y/o M with history of drug use | 20/07/2023 | 28/07/2023 | Stourport, UK | Femoral Blood | - | **2.2** | - | - | Acetaminophen, Benzoylecgonine **(1300)**, Caffeine, Cocaine **(130)**, Codeine **(21)**, Cotinine, Diazepam **(42)**, Hydromorphone **(1.1)**, Morphine **(250)**, Nicotine, Norcocaine, Nordiazepam **(58)**, Noscapine, Papaverine, Phenacetin, Temazepam |
| **78** | 61 y/o white F, found in unlocked car. Decedent seen on hotel security camera driving into lot, pulled into spot, backed up into bush, pulled back into spot (x2). Decedent was found by guest in parking lot. Driver window down at emergency medical services arrival - keys in ‘on’ position, car not running. Past medical history: methamphetamine abuse, tobacco use. | 29/07/2023 | 01/08/2023 | Knoxville, TN | Subclavian Blood | - | **Positive** | - | - | 4-ANPP **(4.8)**, Amphetamine **(510)**, Caffeine, Carboxyhemoglobin **(6 % Saturation)**, Cocaine, Cotinine, Diphenhydramine **(62)**, Fentanyl **(27)**, Gabapentin **(5.1),** Methamphetamine **(1800)**, Morphine **(11)**, Nicotine, Norfentanyl **(26)**, Paroxetine **(39)**, Quinine, Xylazine **(92)** |
| **79** | 20 y/o M, found unresponsive in his fraternity house; reportedly snorted one dose of Quaaludes; extensive drug paraphernalia on scene including cocaine, ketamine, mushrooms, and Kratom | 31/07/2023 | 02/08/2023 | Boulder, CO | Iliac Blood | **Positive** | - | - | - | 2-Fluoro-2-oxo-PCE, Caffeine, Ketamine **(43)**, Methamphetamine, Norketamine **(50)** |
| **80** | 39 y/o M, accidental death | 25/08/2023 | 09/09/2023 | Birmingham, UK | Femoral Blood | - | **Positive** | - | - | Cocaine, Diazepam **(58)**, Diphenhydramine, Naloxone, Nordiazepam **(59)** |
| **81** | 28 y/o M, suspected OD | 15/09/2023 | 16/09/2023 | Albany, NY | Cardiac Blood | - | **Positive** | - | - | 4-ANPP **(0.28)**, 11-Hydroxy Delta-9 THC **(2.6)**, Delta-9 Carboxy THC **(40)**, Delta-9 THC **(6.9)**, Fentanyl **(3.4)**, Naloxone, Norfentanyl **(1.9)**, Xylazine **(12)** |
| **82** | None provided | Not provided | 22/09/2023 | Colorado Springs, CO | Femoral Blood | - | **Positive** | - | - | Bromazolam **(81)** |
| **83** | 38 y/o M | 27/09/2023 | 02/10/2023 | Birmingham, UK | Femoral Blood | - | **6.5** | - | - | 6-Monoacetylmorphine **(12)**, Acetaminophen, Caffeine, Cocaine, Codeine **(41)**, Cotinine, Diazepam **(96)**, Ethanol **(27 mg/dL)**, Hydrocodone, Hydromorphone **(13)**, Mirtazapine, Morphine **(930)**, Nordiazepam **(240)**, Noscapine, Papaverine, Phenacetin, Temazepam, Xylazine **(26)** |
| **84** | 34 y/o M | 12/10/2023 | 16/10/2023 | Birmingham, UK | Femoral Blood | - | **8.3** | - | - | Acetaminophen, Caffeine, Cotinine, Hydromorphone **(1.5),** Mirtazapine**,** Morphine **(120)** |
| **85** | Found with blue pill labeled “B707” | Not provided | 21/11/2023 | Philadelphia, PA | Heart Blood | - | **Positive** | - | - | 4-ANPP, Benzoylecgonine, Bromazolam, Cocaine, Fentanyl, Flubromazepam, Levamisole, Lidocaine, Norcocaine, Norfentanyl, Noroxycodone, Oxycodone, Quinine, Xylazine |

**Key:** M – Male, F – Female, NY – New York, TX – Texas, NJ – New Jersey, PA – Pennsylvania, TN – Tennessee, CT – Connecticut, CO – Colorado, UK – United Kingdom, OD – overdose, HIV – human immunodeficiency virus, THC –tetrahydrocannabinol, 4-ANPP – 4-anilino-*N*-phenethylpiperidine, 2-Fluoro-2-oxo-PCE – 2-(ethylamino)-2-(2-fluorophenyl)cyclohexan-1-one, EDDP – 2-ethylidene-1,5-dimethyl-3,3-diphenylpyrrolidine

Note: All quantitative results pertaining to the additional toxicology findings were obtained using routine methods at NMS Labs. Findings for opioids of the morphinan class are reported as aglycon concentrations. Variations in the inclusion of case descriptors such as age, sex, ethnicity, and circumstances are reflective of the available information for each case provided by medical examiners and coroners.

# ***References***

Cannaert A, Vasudevan L, Friscia M, et al (2018) Activity-based concept to screen biological matrices for opiates and (synthetic) opioids. Clin Chem 64:1221–1229. https://doi.org/10.1373/clinchem.2018.289496

Tsai M-HM, Chen L, Baumann MH, et al (2024) In vitro functional profiling of fentanyl and nitazene analogs at the μ-opioid receptor reveals high efficacy for Gi protein signaling. ACS Chem Neurosci 15:854–867. https://doi.org/10.1021/acschemneuro.3c00750

Vandeputte MM, Cannaert A, Stove CP (2020) In vitro functional characterization of a panel of non-fentanyl opioid new psychoactive substances. Arch Toxicol 94:3819–3830. https://doi.org/10.1007/s00204-020-02855-7

Vandeputte MM, Persson M, Walther D, et al (2022a) Characterization of recent non-fentanyl synthetic opioids via three different in vitro µ-opioid receptor activation assays. Arch Toxicol 96:877–897. https://doi.org/10.1007/s00204-021-03207-9

Vandeputte MM, Van Uytfanghe K, Layle NK, et al (2021) Synthesis, chemical characterization, and μ-opioid receptor activity assessment of the emerging group of “nitazene” 2-benzylbenzimidazole synthetic opioids. ACS Chem Neurosci 12:1241–1251. https://doi.org/10.1021/acschemneuro.1c00064

Vandeputte MM, Vasudevan L, Stove CP (2022b) In vitro functional assays as a tool to study new synthetic opioids at the μ-opioid receptor: Potential, pitfalls and progress. Pharmacol Ther 235:108161. https://doi.org/10.1016/j.pharmthera.2022.108161

Vasudevan L, Vandeputte M, Deventer M, et al (2020) Assessment of structure-activity relationships and biased agonism at the Mu opioid receptor of novel synthetic opioids using a novel, stable bio-assay platform. Biochem Pharmacol 177:113910. https://doi.org/10.1016/j.bcp.2020.113910
